# Hypervalent iodine-guided electrophilic substitution: *para*-selective substitution across aryl iodonium compounds with benzyl groups

**DOI:** 10.3762/bjoc.14.91

**Published:** 2018-05-14

**Authors:** Cyrus Mowdawalla, Faiz Ahmed, Tian Li, Kiet Pham, Loma Dave, Grace Kim, I F Dempsey Hyatt

**Affiliations:** 1 Department of Chemistry and Biochemistry, Adelphi University, 1 South Ave., Garden City, NY, 11530, USA

**Keywords:** electrophilic aromatic substitution, hypernucleofugality, hypervalent iodine, iodonio-Claisen, transmetallation

## Abstract

The reactivity of benzyl hypervalent iodine intermediates was explored in congruence with the reductive iodonio-Claisen rearrangement (RICR) to show that there may be an underlying mechanism which expands the reasoning behind the previously known C–C bond-forming reaction. By rationalizing the hypervalent iodine’s metal-like properties it was concluded that a transmetallation mechanism could be occurring with metalloid groups such as silicon and boron. Hypervalent iodine reagents such as Zefirov’s reagent, cyclic iodonium reagents, iodosobenzene/BF_3_, and PhI(OAc)_2_/BF_3_ or triflate-based activators were tested. A desirable facet of the reported reaction is that iodine(I) is incorporated into the product thus providing greater atom economy and a valuable functional group handle for further transformations. The altering of the RICR’s *ortho*-selectivity to form *para*-selective products with benzyl hypervalent iodine intermediates suggests a mechanism that involves hypervalent iodine-guided electrophilic substitution (HIGES).

## Introduction

**Scheme 1:**
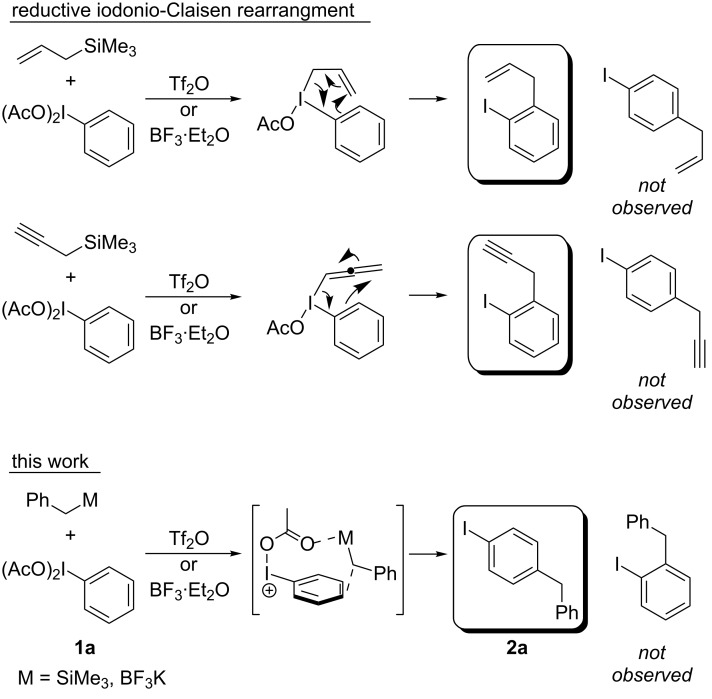
Examples of the reductive iodonio-Claisen rearrangement compared to new reactivity seen with benzyl metalloids.

Hypervalent iodine compounds have been known for over a hundred years, but it was not until their renaissance in the 1990’s that many of these useful reagents became a staple in synthetic chemistry laboratories [1–2]. Although hypervalent iodine reagents are commonly used in oxidation reactions, they have also found their own niche in useful C–C bond-formation and C–H activation reactions [3–5]. One such C–C bond formation (Scheme 1) was discovered by Oh and co-workers in 1988, and although it was based on previous work by the Ochiai group, the paper was the first to suggest a six-membered transition state indicative of a Claisen reaction [6–7]. In 1991, Ochiai and co-workers coined the phrase reductive iodonio-Claisen rearrangement (RICR) to describe the product selectivity they encountered [8].

More recently, exceptional progress has been made in investigating the RICR’s substrate scope (electron-donating versus electron-withdrawing substituents on PhI(OAc)_2_ (**1a**)), mechanism (deuterium labelling studies), product yields and selectivities based on appropriate solvents, temperatures, and Lewis acids [9–11]. A recent digest of RICR theorized an underlying mechanistic concept dubbed iodine-guided electrophilic aromatic substitution (IGEAS) [12]; the basis for which the work herein is titled. Other related work focused on the activation of hypervalent iodine [13], and a computational study that suggested a concerted iodination/deprotonation (CID) that is analogous to concerted metallation/deprotonation (CMD) for cationic hypervalent iodine [14]. These studies and others on the electrophilic nature and metal-like properties of iodine(III) were particularly significant in the development of the reaction reported in this communication [15–16].

A commonality in the RICR is that the proposed mechanisms involve an unstable allyl or propargyl hypervalent iodine intermediate. To the best of our knowledge, no allyl, propargyl, or alkyl hypervalent iodine species have been isolated at room temperature (besides fluorinated alkyl chains [17]), thus trapping an intermediate to validate the mechanism seemed unlikely. The process in which these hypervalent iodine intermediates form from metalloid substituted substrates has not been fully explored. It has been suggested that there may be a more all-inclusive iodine-guided mechanism that could account for a wider range of hypervalent iodine reactivity [12]. To this end, it is theorized that hypervalent iodine’s metal-like properties allow it to undergo transmetallation with an appropriate metalloid substrate. This concept is counter to previous mechanisms in which the electrophilic hypervalent iodine reagent is attacked by unsaturated C–C bonds [18–22]. To show evidence for this transmetallation event, benzyl metalloid groups were used under the same reaction conditions as the RICR. The resulting diphenylmethane structure obtained after the C–C bond formation could have relevant medicinal applications since it is the core of many marketed pharmaceutical drugs with antihistaminic and anxiolytic properties [23–26].

## Results and Discussion

In the reaction with PhI(OAc)_2_ (**1a**), 0.5 equiv triflic anhydride, and BnM (where M is TMS or BF_3_K), a 72% yield of a coupled product was isolated, but the connection was unexpectedly at the *para-* not the *ortho*-position as the RICR might have predicted (Table 1). Further experiments of this hypervalent iodine-guided electrophilic substitution (HIGES) reaction were performed by varying the hypervalent iodine starting material, the activator, the solvent, and the temperature at which the activated hypervalent iodine reagent formed (Table 1). Varying the temperature at 25 °C, 0 °C, and −50 °C did not cause a substantial difference in yield, but at −50 °C the solubility of the activated hypervalent iodine species seemed poor upon visual inspection. It should be noted that the only other major products in the resultant reaction mixture were the decomposition of PhI(OAc)_2_ (**1a**) or PhIO to PhI, and unreacted BnTMS or BnBF_3_K. The results in Table 1 show that silyl groups seem to be superior to boron groups for the reaction to afford a good yield. The solvent choices were made based on low nucleophilicity and polarity being high enough to dissolve the hypervalent iodine starting material. Surprisingly, the protic solvent methanol worked to synthesize the product albeit in low yield.

**Table 1:** Optimization of HIGES reaction.^a^

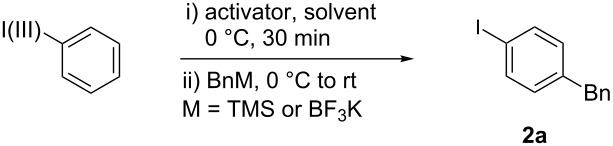					

Entry	Metalloid (M)	I(III) Reactant	Activator	Solvent	Yield (%)

1	TMS	PhI(OAc)_2_	Tf_2_O (0.5 equiv)	CDCl_3_	72
2	TMS	PhI(OAc)_2_	Tf_2_O (1.0 equiv)	CDCl_3_	73
3	TMS	PhI(OAc)_2_	BF_3_·Et_2_O (1.0 equiv)	CDCl_3_	64
4	TMS	PhIO	BF_3_·Et_2_O (1.0 equiv)	CDCl_3_	80
5	TMS	PhIO	BF_3_·Et_2_O (1.0 equiv)	MeOH	13
6	TMS	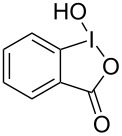 **3**	Tf_2_O (1.0 equiv)	DCM	0
7	TMS	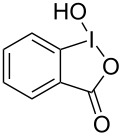 **3**	Tf_2_O (1.0 equiv)	CH_3_CN	0
8	BF_3_K	PhI(OAc)_2_	Tf_2_O (1.0 equiv)	CH_3_CN	17
9	BF_3_K	PhI(OAc)_2_	Tf_2_O (0.5 equiv)	CH_3_CN	24
10	BF_3_K	PhI(OAc)_2_	BF_3_·Et_2_O (1.0 equiv)	CDCl_3_	24
11	BF_3_K	PhIO	BF_3_·Et_2_O (1.0 equiv)	CDCl_3_	43
12	BF_3_K	PhIO	Tf_2_O (0.5 equiv)	CH_3_CN	18
13	TMS	PhI(OAc)_2_	none	CDCl_3_	0

^a^All reactions used 0.055 M to 0.115 M I(III) reactant with the specified solvent and activator at 0 °C for 30 min followed by the addition of 1.0 equiv of BnTMS or BnBF_3_K and allowing the mixture to warm to room temperature. Isolated yields are reported.

Another result is that by changing the metalloid substrate to BnBF_3_K (Table 1, entries 8–12), the reaction proceeds to give a product with generally lower yield. This fact is potentially explained by the partial solubility of trifluoroborates in the solvents used. The displacement of silyl and boron groups with hypervalent iodine seems to suggest that a transmetallation event from Si/B to I is occurring rather than an addition reaction to the unsaturation of the benzyl group followed by elimination of the metalloid group. The latter of which would be consistent with the mechanism currently theorized for making alkenyl and alkynyl hypervalent iodine species [18–22].

While investigating the substrate scope it was found that the substitution pattern is dependent upon the substituents on the I(III) reactant (Table 2). The trend of *para*-substitution to specific substituents appears to provide some evidence for a similar intermediate that relates to each reaction (for instance, Table 1, entry 1 compared to entry 8 or 9). Also, the optimization study showed comparable yields for activating reagents yet the use of BF_3_·Et_2_O seemed to be superior to Tf_2_O in the case of some I(III) reactants.

**Table 2:** Substrate scope of substituted hypervalent iodine species.

Entry	I(III) Reactant	Product	Yield^a^ (%)

1	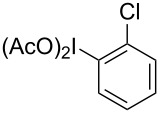 **1b**	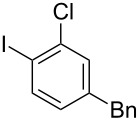 **2b**	2, 15^b,c^
2	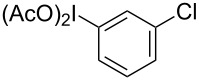 **1c**	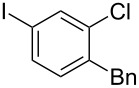 **2c**	6, 23^b,c^
3	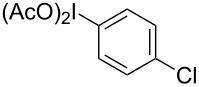 **1d**	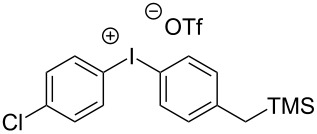 **2d**	23
4	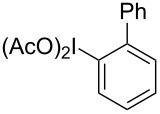 **1e**	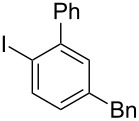 **2e**	25
5	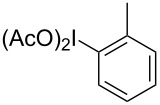 **1f**	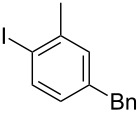 **2f**	52
6	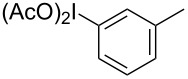 **1g**	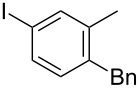 **2g**	45
7	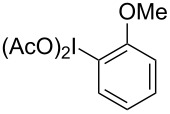 **1h**	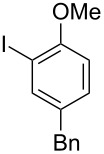 **2h**	28
8	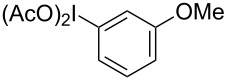 **1i**	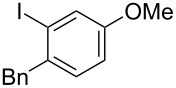 **2i**	4, 50^b^
9	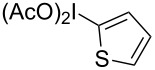 **1j**	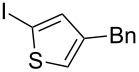 **2j**	76
10	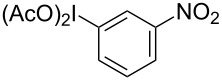 **1k**	NA	0
11	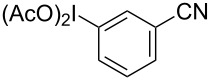 **1l**	NA	0

^a^All reactions used 0.055 M to 0.115 M I(III) reactant with 0.5 equiv of Tf_2_O in CH_2_Cl_2_ or CDCl_3_ at 0 °C for 30 minutes followed by 1.0 equiv of BnTMS unless otherwise specified. Isolated yields are reported. ^b^Reaction used 1.0 equiv BF_3_·Et_2_O as an activator instead of Tf_2_O. ^c^NMR yield in CDCl_3_.

In all the reactions tested, there were no products formed with the *ortho*-substitution pattern in contrast to the RICR, however, it is clear that electron-donating groups influence the ring substitution. When weaker electron-donating and electron-withdrawing groups are used (Table 2, entries 1, 2, 4, 5 and 6), the C–C bond formation occurs *para* to the iodine, but when strong electron-donating groups are used (Table 2, entries 7 and 8) then the *para*-selectivity is dictated by that group. The effect of electron donation is also demonstrated in the thiophene (**1j**) group tested (Table 2, entry 9). In the case of electron-withdrawing groups, deactivation occurs and the only reaction is the decomposition of the I(III) to I(I) (Table 2, entries 10 and 11). The fact that no product was formed when using electron-withdrawing groups could also explain how the carbonyl of the cyclic iodonium (**3**) caused that specific reagent to fail (Table 1, entries 6 and 7). Several *para*-substituted I(III) reactants were attempted but all led to complex mixtures with the exception of the diaryliodonium triflate **2d** (Table 2, entry 3). These key features in the substitution pattern of the substrate scope led to experiments where tests were performed to better understand, if the HIGES reaction is a concerted mechanism, like RICR, or a stepwise process.

**Scheme 2:**
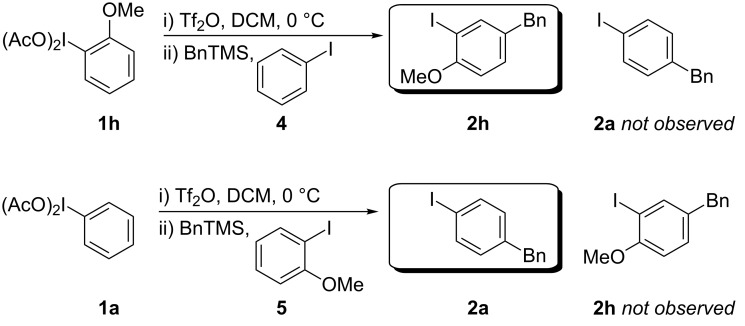
Crossover reaction experiments.

In the article and further in a footnote of Khatri and Zhu’s publication [11] compelling evidence was shown by deuterium labelling studies supporting a concerted intramolecular mechanism (RICR) occurring rather than a stepwise intermolecular one. To corroborate their findings with our own, we investigated the HIGES reaction through crossover experiments which appear to conclude a concerted mechanism is occurring (Scheme 2). Two crossover reactions were performed for both iodobenzene (**4**) and 2-iodoanisole (**5**) to prevent substrate bias. One stepwise mechanism that the results in Scheme 2 eliminate is the possibility that the I(III) is decomposing to I(I) and then subsequently reacts in an electrophilic substitution on the transmetallated benzyl hypervalent iodine intermediate. If such a stepwise mechanism were to occur then one would expect to see an additional coupling product.

**Scheme 3:**
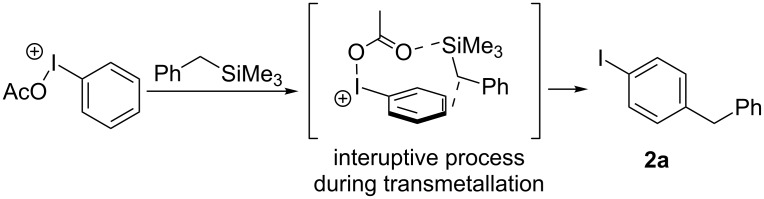
Suggested mechanism based on product formation and crossover experiments.

**Scheme 4:**
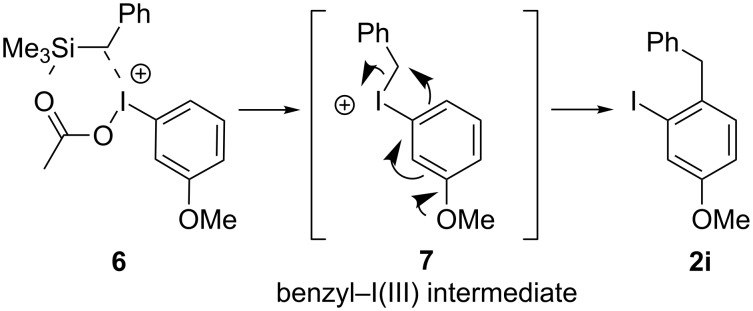
Proposed mechanism for the generation of **2i** from Table 2, entry 8.

Based on the evidence provided by these crossover reactions it rules out such a stepwise reaction thus we propose a concerted mechanism. The mechanism speculated in Scheme 3 shows a concerted demetallation of the metalloid as the C–C bond is forming. Before the transmetallation of the metalloid group, an interruptive process could be occurring that provides an orbital overlap at the benzylic methylene and the *para-*position of the aryl iodine. In the case of entry 7 from Table 2, the methoxy group of **1h** directs the *para-*positioning in a similar manner, yet entry 8 from Table 2 would likely result from a different intermediate (Scheme 4).

In Scheme 4, a mechanism where a C–C bond forms by an intramolecular attack on a benzylic methylene with the hypernucleofuge attached (**6**). The mechanism in Scheme 4 shows the transmetallation occuring instead of being interrupted as in Scheme 3. The combination of both mechanisms (Scheme 3 and Scheme 4) explains how substitution *ortho* to the iodine (*para* to the methoxy group) could be generated from a benzyl–I(III) intermediate (**7**), while the products found from the interruptive transmetallation process obey the mechanism shown in Scheme 3. Another point to note is that since a product can form *ortho* to the iodine, sterics are unlikely the sole cause of the *para*-selectivity of the other reactions shown. Further investigation of in situ generated benzyl–I(III) and alkyl–I(III) intermediates is still being conducted in the research group.

## Conclusion

Towards the goal of establishing a robust, novel methodology utilizing hypervalent iodine in C–C bond formation, a hypervalent iodine-guided electrophilic substitution (HIGES) reaction was discovered. The new reaction is thought to have some similarities to the reductive iodonio-Claisen rearrangement except that, with respect to the iodine, *para*-substituted instead of *ortho*-substituted products are mainly isolated. Future directions seek to elucidate the mechanism of the HIGES reaction and to develop the methodology into a reaction capable of synthesizing a variety of diphenylmethane structures.

## Experimental

General procedure: The hypervalent iodine reagent (1.0 equiv) was added to the appropriate solvent. The reaction was cooled to 0 °C and the appropriate activator (0.5 or 1.0 equiv) added before allowing the reaction to stir for 30 min. The metalloid reagent, BnTMS or BnBF_3_K (1.0 equiv), was then added and allowed to warm to room temperature. The reaction mixtures were stirred for a period of 10 min to 2 h while monitoring the progress by TLC. The products were purified through PREP-TLC (hexane/ethyl acetate 90:10).

## Supporting Information

File 1 Synthetic procedures, characterization data and copies of spectra.
