# Supplementary material for: Hypervalent iodine-guided electrophilic substitution: para-selective substitution across aryl iodonium compounds with benzyl groups
Source: Beilstein J Org Chem. 2018 May 14;14:1039–45. doi: 10.3762/bjoc.14.91 (PMC6009249; doi:10.3762/bjoc.14.91)
Supplement: File 1 — Synthetic procedures, characterization data and copies of spectra. [file Beilstein_J_Org_Chem-14-1039-s001.pdf]

Supporting Information

for

Hypervalent iodine-guided electrophilic substitution: *para*-selective substitution across aryl iodonium compounds with benzyl groups

Cyrus Mowdawalla, Faiz Ahmed, Tian Li, Kiet Pham, Loma Dave, Grace Kim, I. F. Dempsey Hyatt*

Address: Department of Chemistry and Biochemistry, Adelphi University, 1 South Ave., Garden City, NY, 11530, USA

Email: Ivan Fabe Dempsey Hyatt - ihyatt@adelphi.edu

* Corresponding author

Synthetic procedures, characterization data and copies of spectra

Table of Contents:

1. General information S2
2. General procedure S2
3. Characterization data of compounds S3
4. $^{1}H/^{13}C$ NMR spectra S7
5. References S29

1. General Information

All anhydrous reactions were performed in oven-dried glassware under a nitrogen atmosphere. Unless otherwise noted, all solvents and reagents were obtained from commercial sources and used without further purification. Compounds **1b**[1], **1c**[2], **1d**[3], **1f**[1], **1g**[1], **1h**[4], **1i**[5], **1j**[6], **1k**[7], **1l**[8], benzyltrimethylsilane[9], and benzyltrifluorborate[10] were synthesized from their corresponding references, and their NMR data matched what was previously reported. NMR yields were obtained by using dioxane as an internal standard in a $CDCl_3$ solution. High resolution mass spectra were acquired on a Thermo Fisher Scientific LTQ Orbitrap XL MS system. A Bruker Avance III 500 MHz spectrometer was used to record the ^{1}H and ^{13}C NMR spectra in $CDCl_3$ and ACN-d_3. ^{1}H NMR data is reported as chemical shift (δ, ppm), multiplicity (s, singlet; d, doublet; t, triplet; q, quartet; m, multiplet), integration, and coupling constant (Hz), while ^{13}C is reported as chemical shift (δ, ppm). Preparatory thin layer chromatography (PREP-TLC) was completed using SillaPlate TLC: Glass Backed TLC Extra Hard Layer (60 Å, 250 µm); infused with a fluorescent indicator 254 nm.

2. General procedure

The hypervalent iodine reagent (1 equiv) was added to the appropriate solvent and cooled to 0 °C. Then, the appropriate activator (0.5 or 1.0 equiv) was added and stirring continued for 30 min. The metalloid reagent, BnTMS or $BnBF_3K$, (1.0 equiv) was then added and the mixture allowed to warm to room temperature. The mixture was stirred for a period of 10 min to 2 h while monitoring the progress of the reaction by TLC. The product was purified through PREP-TLC (hexane/ethyl acetate 90:10).

3. Characterization data of compounds

[1,1'-Biphenyl]-2-yl-iodanediyl diacetate (1e)

The following compound, **1e**, was prepared by the reference provided.[4]

From 2-iodobiphenyl (1.00 g, 3.57 mmol), glacial acetic acid (45 mL), sodium perborate tetrahydrate (3.56 g, 35.7 mmol), (**1e**) was obtained in 53% (0.561 mg).

^{1}H NMR (500 MHz, $CDCl_3$): δ 8.35 (dd, J = 1.1, 8.0 Hz, 4 H), 7.68 (dd, J = 1.1, 7.4 Hz, 4 H), 7.63 - 7.60 (m, 4 H), 7.47 - 7.42 (m, 26 H), 1.93 (s, 6 H).

^{13}C NMR (125 MHz, $CDCl_3$): δ 176.2, 145.6, 141.7, 137.5, 132.4, 130.8, 129.6, 129.0, 128.7, 128.3, 125.9, 20.3.

1-Benzyl-4-iodobenzene (2a)

From iodobenzenediacetate (17.7 mg, 0.055 mmol), deuterated chloroform (1 mL), trifluoromethanesulfonic anhydride (9.28 µL, 0.055 mmol), and benzyltrimethylsilane (9 uL, 0.055 mmol), (**2a**) was obtained in 73% (10.1 mg) yield.

^{1}H NMR (500 MHz, $CDCl_3$): δ 7.63 - 7.59 (m, 2 H), 7.32 - 7.28 (m, 2 H), 7.24 - 7.20 (m, 1 H), 7.18 - 7.15 (m, 2 H), 6.97 - 6.93 (m, 2 H), 3.93 (s, 2 H)

^{13}C NMR (125 MHz, $CDCl_3$): δ 140.8, 137.5, 131.0, 128.8, 128.6, 128.5, 126.3, 41.4, 31.9, 29.7, 29.4, 22.7, 14.1, 0.0

HRMS (APCI): calcd. for $[C_{13}H_{11}I]^+$: 293.9905, found: 293.9907.

4-Benzyl-2-chloro-1-iodobenzene (2b)

From (2-chlorophenyl)iodanediyl diacetate (**1b**, 0.204 mg, 0.574 mmol), dry chloroform (5 mL), trifluoromethanesulfonic anhydride (50 µL, 0.30 mmol), and benzyltrimethylsilane (100 µL, 0.610 mmol), (**2b**) was obtained in 2% (4.2 mg) yield.

^{1}H NMR (500 MHz, $CDCl_3$): δ 7.75 (d, J = 8.2 Hz, 1 H), 7.31 (t, J = 1.0 Hz, 2 H), 7.29 (d, J = 1.9 Hz, 2 H), 7.25 (d, J = 1.0 Hz, 1 H), 7.16 (dd, J = 0.9, 7.9 Hz, 2 H), 6.79 (t, J = 1.0 Hz, 1 H), 3.91 (s, 2 H).

^{13}C NMR (125 MHz, $CDCl_3$): δ 143.2, 140.1, 139.6, 129.8, 128.8, 128.7, 128.7, 126.6, 94.9, 41.1, -0.6

HRMS (APCI): calcd. for $[C_{13}H_{10}ClI]^+$: 327.9516, found: 327.9514.

1-Benzyl-2-chloro-4-iodobenzene (2c)

From (3-chlorophenyl)iodanediyl diacetate (**1c**, 0.189 g, 0.305 mmol), dry chloroform (5 mL), trifluoromethanesulfonic anhydride (25 µL, 0.15 mmol), and benzyltrimethylsilane (50 µL, 0.305 mmol), (**2c**) was obtained in 5.7 mg or 6%.

^{1}H NMR (500 MHz, $CDCl_3$): δ 7.73 (d, J = 1.6 Hz, 1 H), 7.50 (dd, J = 1.9, 8.2 Hz, 1 H), 7.31 (t, J = 7.4 Hz, 2 H), 7.23 (t, J = 7.4 Hz, 1 H), 7.17 (d, J = 7.4 Hz, 2 H), 6.87 (d, J = 7.9 Hz, 1 H), 4.05 (s, 2 H).

^{13}C NMR (125 MHz, $CDCl_3$): δ 138.8, 138.6, 137.7, 135.9, 135.2, 132.4, 128.9, 128.6, 126.5, 91.1, 38.8.

HRMS (APCI): calcd. for $[C_{13}H_{10}ClI]^+$: 327.9516, found: 327.9518.

(4-Chlorophenyl)(4-((trimethylsilyl)methyl)phenyl)iodonium triflate (2d)

From (4-chlorophenyl)iodanediyl diacetate (**1d**, 0.0562 g, 0.165 mmol), dry dichloromethane (3 mL), trifluoromethanesulfonic anhydride (13.92 µL, 0.0825 mmol), and benzyltrimethylsilane (27 µL, 0.165 mmol), (**2d**) was obtained in 23% (15.2 mg) yield.

^{1}H NMR (500 MHz, $CDCl_3$): δ 7.91 (d, J = 8.5 Hz, 2 H), 7.84 (d, J = 8.2 Hz, 2 H), 7.39 (d, J = 8.5 Hz, 2 H), 7.06 (d, J = 7.9 Hz, 2 H), 2.14 (s, 2 H), -0.01 - -0.03 (m, 9 H)

^{13}C NMR (125 MHz, $CDCl_3$): δ 147.6, 139.3, 136.1, 135.4, 132.2, 131.7, 110.7, 107.4, 30.9, 27.9, -2.2

HRMS (APCI): calcd. for $[C_{16}H_{19}ClISi]^+$: 400.9989, found: 400.9995.

5-Benzyl-2-iodo-1,1'-biphenyl (2e)

From [1,1'-biphenyl]-2-yl-iodanediyl diacetate (**1e**, 23.1 mg, 0.055 mmol), dry dichloromethane (1 mL), trifluoromethanesulfonic anhydride (4.64 µL, 0.0275 mmol), and benzyltrimethylsilane (9 µL, 0.055 mmol), (**1e**) was obtained in 25% (5.1 mg).

^{1}H NMR (500 MHz, $CDCl_3$): δ 7.85 (d, J = 8.2 Hz, 1 H), 7.44 - 7.38 (m, 3 H), 7.34 - 7.28 (m, 5 H), 7.24 - 7.18 (m, 3 H), 7.17 (d, J = 2.2 Hz, 1 H), 6.88 (dd, J = 2.2, 7.9 Hz, 1 H), 3.97 - 3.95 (m, 2 H).

^{13}C NMR (125 MHz, $CDCl_3$): δ 146.6, 144.1, 141.4, 140.3, 139.5, 130.8, 129.5, 129.3, 128.9, 128.6, 127.9, 127.6, 126.3, 95.5, 41.4.

HRMS (APCI): calcd. for $[C_{19}H_{15}I]^+$: 370.0219, found: 370.0217.

4-Benzyl-1-iodo-2-methylbenzene (2f)

From *o*-tolyliodanediyl diacetate (**1f**, 18.6 mg, 0.055 mmol), dry dichloromethane (1 mL), trifluoromethanesulfonic anhydride (4.64 µL, 0.0275 mmol), and benzyltrimethylsilane (9 µL, 0.055 mmol), (**2f**) was obtained in 52% (8.7 mg).

^{1}H NMR (500 MHz, $CDCl_3$): δ 7.72 (d, J = 7.9 Hz, 1 H), 7.30 (d, J = 7.6 Hz, 2 H), 7.25 - 7.22 (m, 1 H), 7.20 - 7.17 (m, 2 H), 7.09 (d, J = 2.2 Hz, 1 H), 6.73 (dd, J = 2.2, 7.9 Hz, 1 H), 3.91 (s, 2 H), 2.41 (s, 3 H).

^{13}C NMR (125 MHz, $CDCl_3$): δ 141.4, 141.3, 138.9, 130.5, 129.6, 128.8, 128.5, 128.2, 126.2, 98.1, 41.3, 28.0.

HRMS (APCI): calcd. for $[C_{14}H_{13}I]^+$: 308.0062, found: 308.0063.

1-Benzyl-4-iodo-2-methylbenzene (2g)

From *m*-tolyliodanediyl diacetate (**1g**, 20.1 mg, 0.055 mmol), dry dichloromethane (1 mL), trifluoromethanesulfonic anhydride (4.64 µL, 0.0275 mmol), and benzyltrimethylsilane (9 µL, 0.055 mmol), (**2g**) was obtained in 45% (7.5 mg).

^{1}H NMR (500 MHz, $CDCl_3$): δ 7.53 (d, J = 1.3 Hz, 1 H), 7.47 (dd, J = 1.7, 8.0 Hz, 1 H), 7.30 - 7.26 (m, 2 H), 7.23 - 7.18 (m, 1 H), 7.10 (dd, J = 0.9, 7.9 Hz, 2 H), 6.84 (d, J = 7.9 Hz, 1 H), 3.93 (s, 2 H), 2.20 (s, 3 H).

^{13}C NMR (125 MHz, $CDCl_3$): δ 139.6, 139.2, 138.9, 138.7, 135.0, 131.8, 128.6, 128.5, 126.1, 91.7, 39.0, 19.3.

HRMS (APCI): calcd. for $[C_{14}H_{13}I]^+$: 308.0062, found: 308.0062.

4-Benzyl-2-iodo-1-methoxybenzene (2h)

From (2-methoxyphenyl)iodanediyl diacetate (**1h**, 0.0562 g, 0.165 mmol), dry dichloromethane (3 mL), trifluoromethanesulfonic anhydride (13.92 µL, 0.0825 mmol), and benzyltrimethylsilane (27 µL, 0.165 mmol), (**2h**) was obtained in 28% (14.9 mg) yield.

^{1}H NMR (500 MHz, $CDCl_3$): δ 7.62 (d, J = 2.2 Hz, 1 H), 7.32 - 7.28 (m, J = 1.0, 1.0 Hz, 2 H), 7.22 (d, J = 7.6 Hz, 1 H), 7.17 (d, J = 6.9 Hz, 2 H), 7.12 (dd, J = 2.0, 8.4 Hz, 1 H), 6.75 (d, J = 8.5 Hz, 1 H), 3.89 (s, 2 H), 3.86 (s, 3 H).

^{13}C NMR (125 MHz, $CDCl_3$): δ 140.8, 139.7, 135.4, 129.9, 128.8, 128.5, 126.2, 110.8, 86.0, 56.4, 40.5.

HRMS (APCI): calcd. for $[C_{14}H_{13}IO]^+$: 324.0011, found: 324.0013.

1-Benzyl-2-iodo-4-methoxybenzene (2i)

From (3-methoxyphenyl)iodanediyl diacetate (**1i**, 30 mg, 0.0852 mmol), dry dichloromethane (1 mL), boron trifluoride diethyl etherate (10.0 µL, 0.0796 mmol), and benzyltrimethylsilane (13.5 µL, 0.0284 mmol), (**2i**) was obtained in 50% (13.7 mg) yield.

^{1}H NMR (500 MHz, ACN-d3): δ 7.28 - 7.23 (m, 4 H), 7.15 - 7.20 (m, 3 H), 6.88 (d, J = 7.7 Hz, 1 H), 3.88 (s, 2 H), 3.78 (s, 3 H).

^{13}C NMR (125 MHz, ACN-d3): δ 158.9, 141.6, 132.8, 130.8, 130.5, 129.6, 129.3, 126.9, 120.9, 92.0, 56.4, 36.0.

HRMS (APCI): calcd. for $[C_{14}H_{13}IO]^+$: 324.0011, found: 324.0014.

2-Benzyl-5-iodothiophene (2j)

From thiophen-2-yl-iodanediyl diacetate (**1j**, 56.1 mg, 0.165 mmol), dry dichloromethane (3 mL), trifluoromethanesulfonic anhydride (14.0 µL, 0.0825 mmol), and benzyltrimethylsilane (27 µL, 0.165 mmol), (**2j**) was obtained in 76% (37.9 mg).

^{1}H NMR (500 MHz, $CDCl_3$): δ 7.34 - 7.30 (m, 2 H), 7.27 - 7.22 (m, 3 H), 7.06 (d, J = 3.8 Hz, 1 H), 6.50 (dd, J = 1.1, 3.6 Hz, 1 H), 4.13 (s, 2 H).

^{13}C NMR (125 MHz, $CDCl_3$): δ 150.4, 139.6, 136.7, 128.6, 128.6, 126.8, 126.7, 70.9, 36.3.

HRMS (APCI): calcd. for $[C_{11}H_{19}IS]^+$: 299.9470, found: 299.9471.

4. $^{1}H/^{13}C$ 2D NMR spectra

[1,1'-Biphenyl]-2-yl-iodanediyl diacetate (1e)

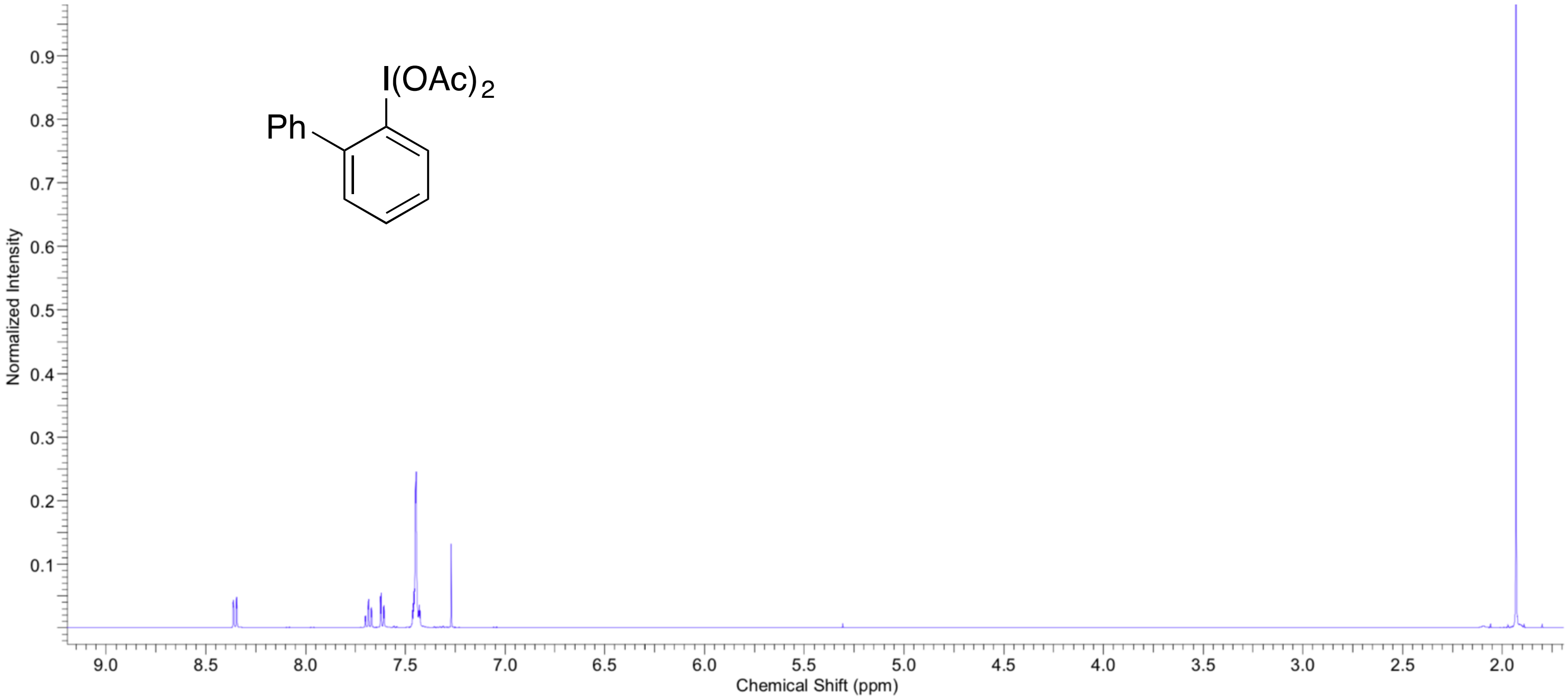

[1,1'-Biphenyl]-2-yl-iodanediyl diacetate (1e)

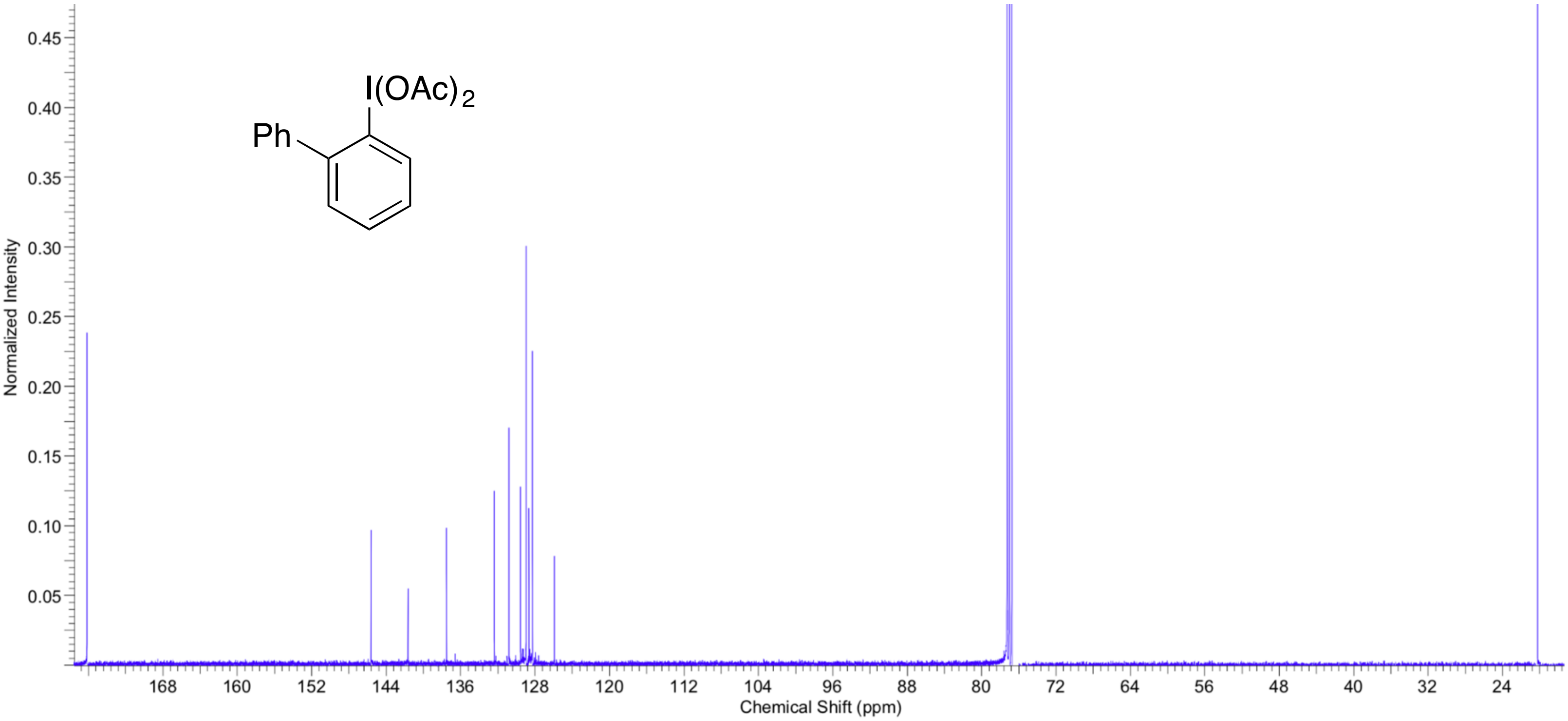

[1,1'-Biphenyl]-2-yl-iodanediyl diacetate (1e)

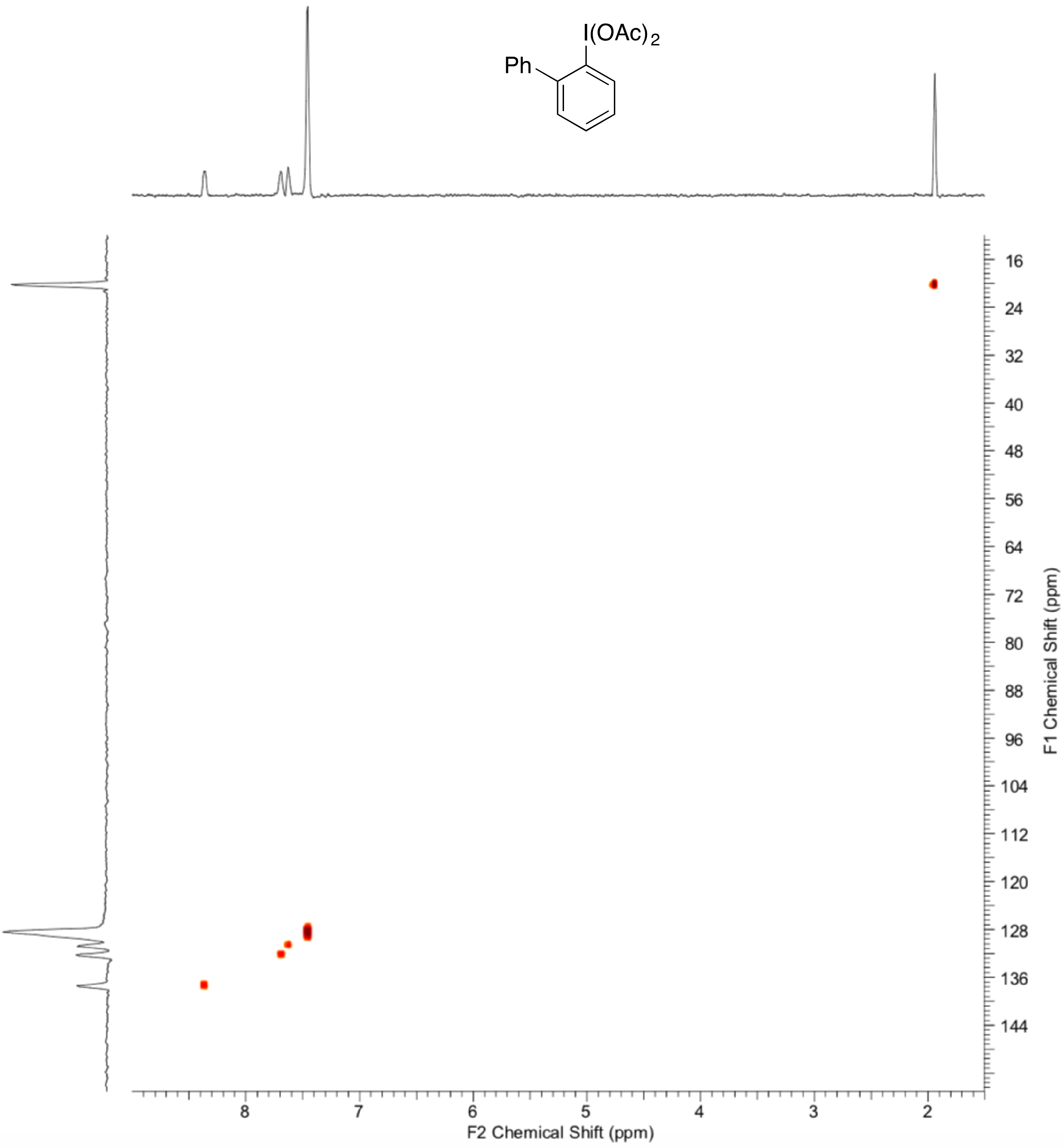

1-Benzyl-4-iodobenzene (2a)

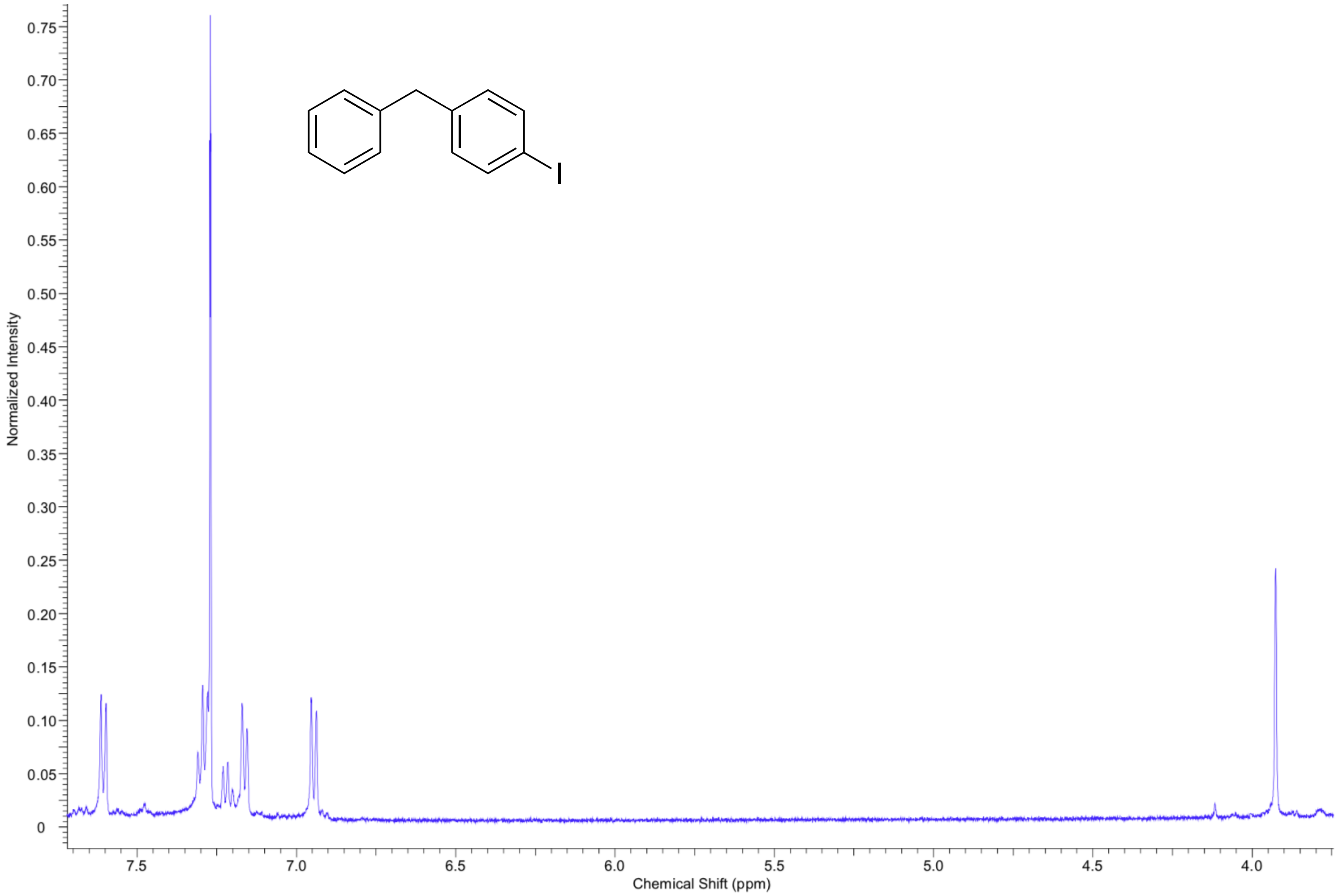

1-Benzyl-4-iodobenzene (2a)

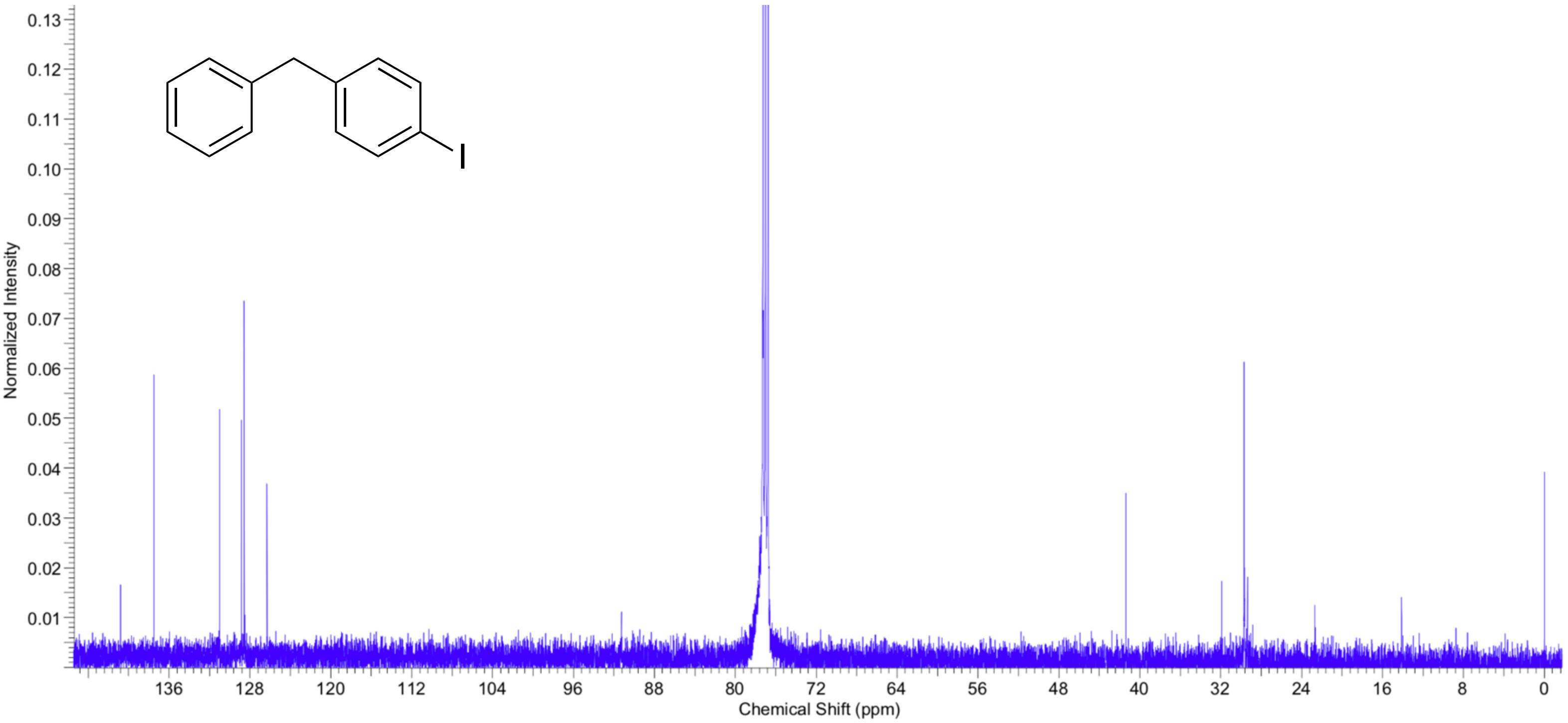

4-Benzyl-2-chloro-1-iodobenzene (2b)

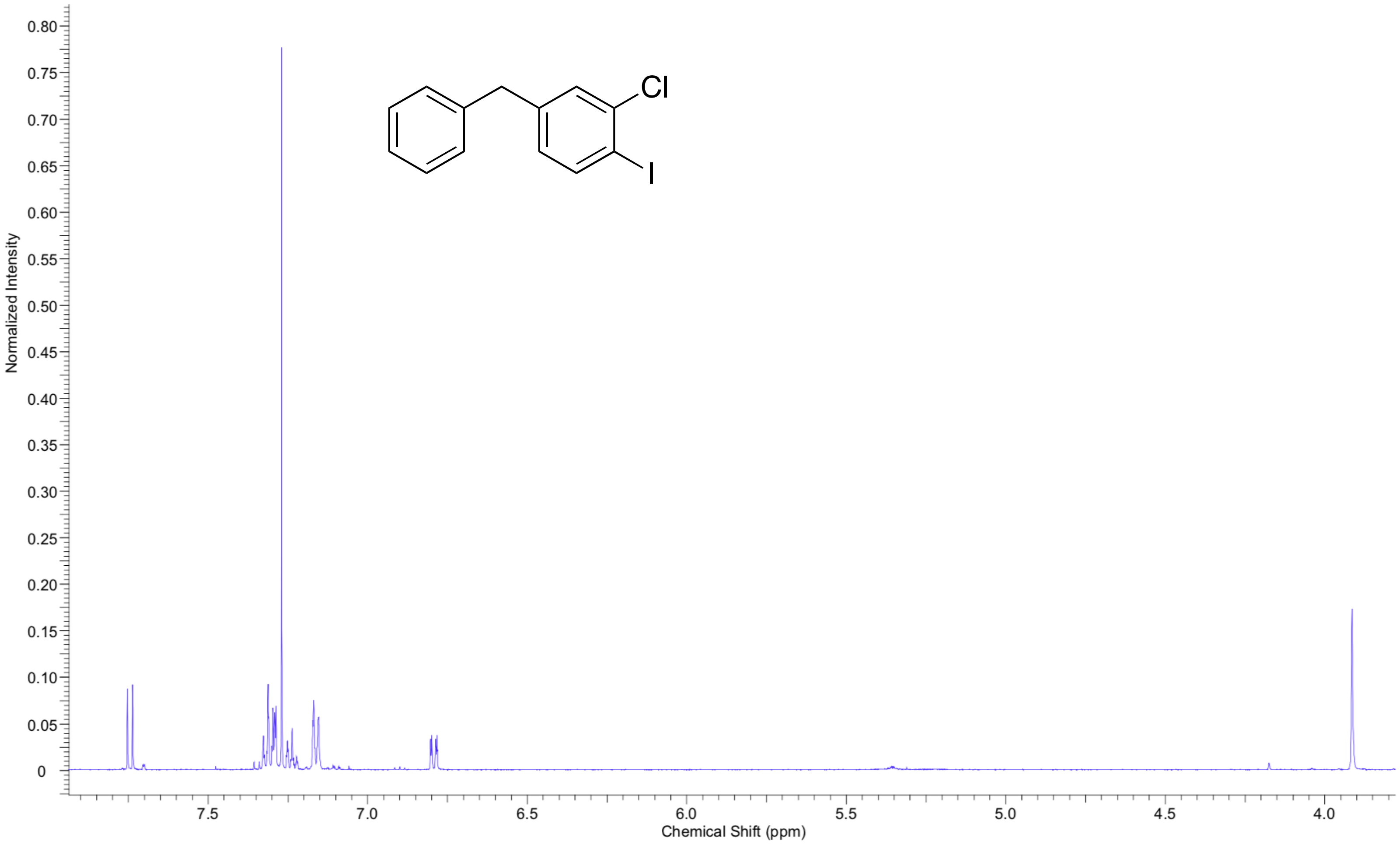

4-Benzyl-2-chloro-1-iodobenzene (2b)

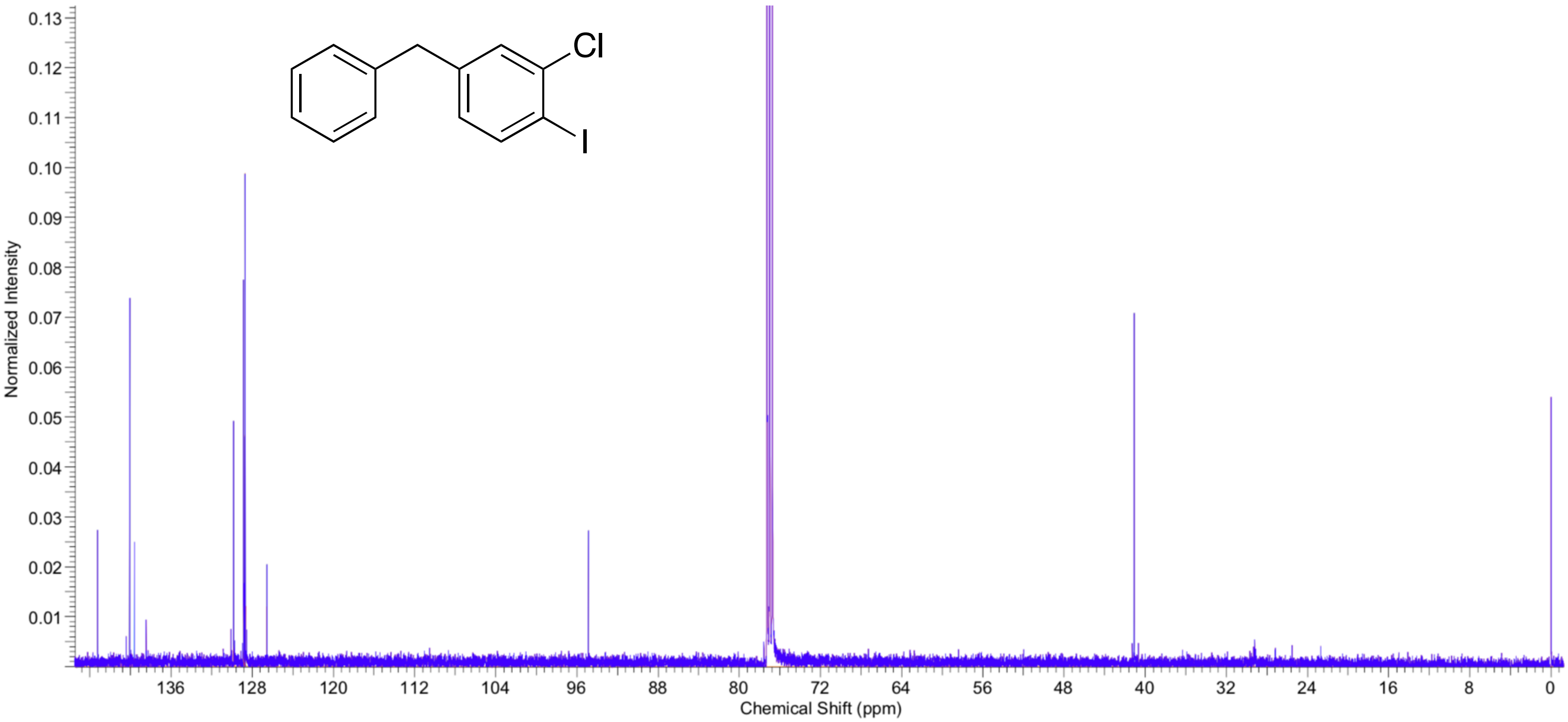

1-Benzyl-2-chloro-4-iodobenzene (2c)

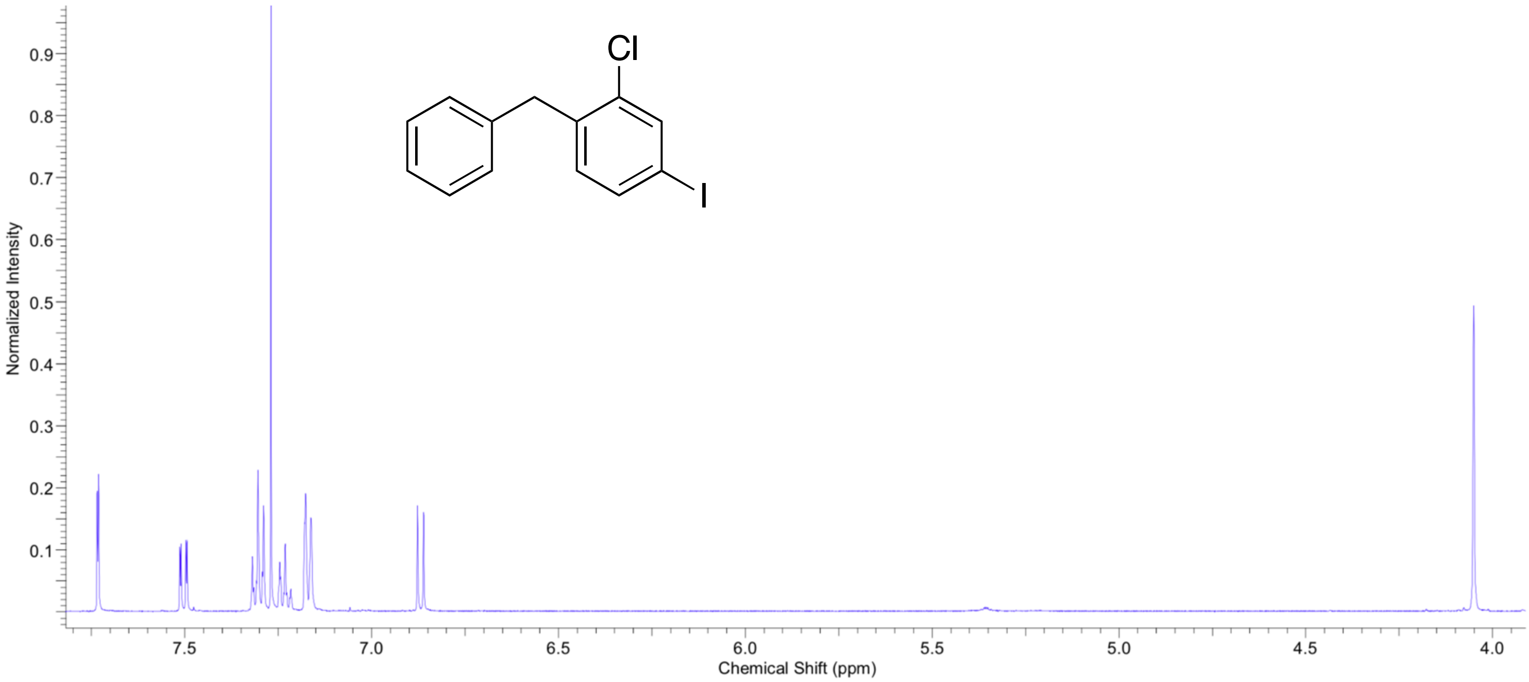

1-Benzyl-2-chloro-4-iodobenzene (2c)

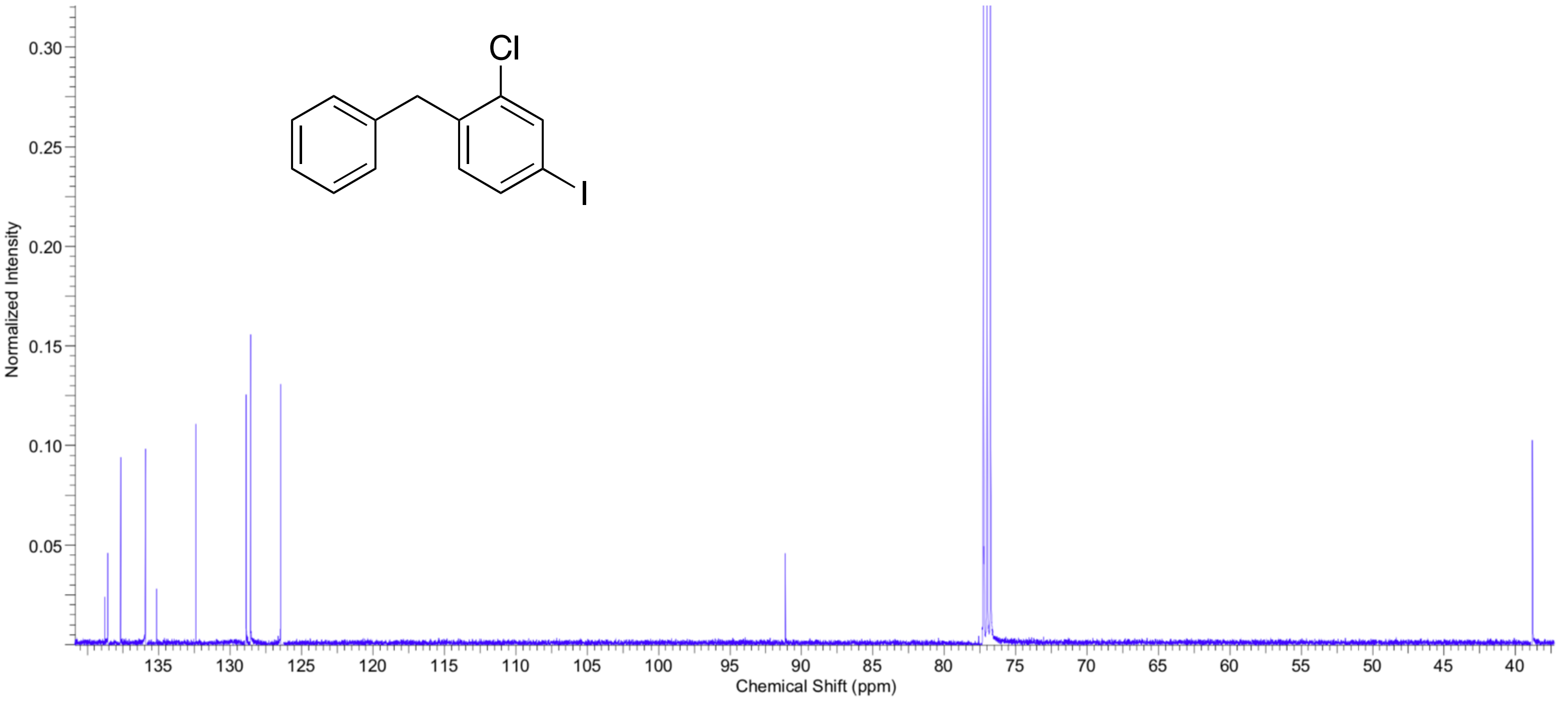

(4-Chlorophenyl)(4-((trimethylsilyl)methyl)phenyl)iodonium triflate (2d)

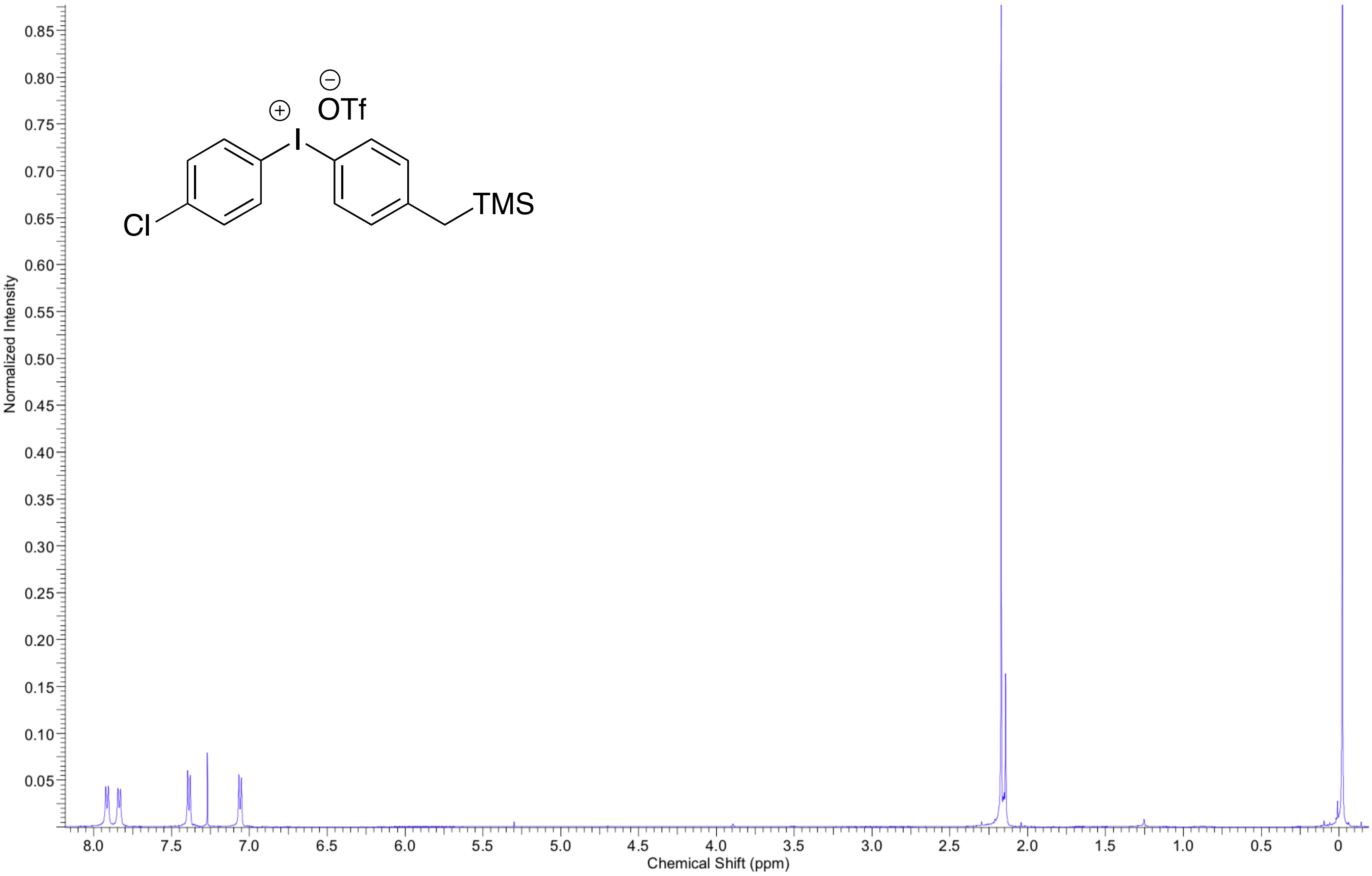

(4-Chlorophenyl)(4-((trimethylsilyl)methyl)phenyl)iodonium triflate (2d)

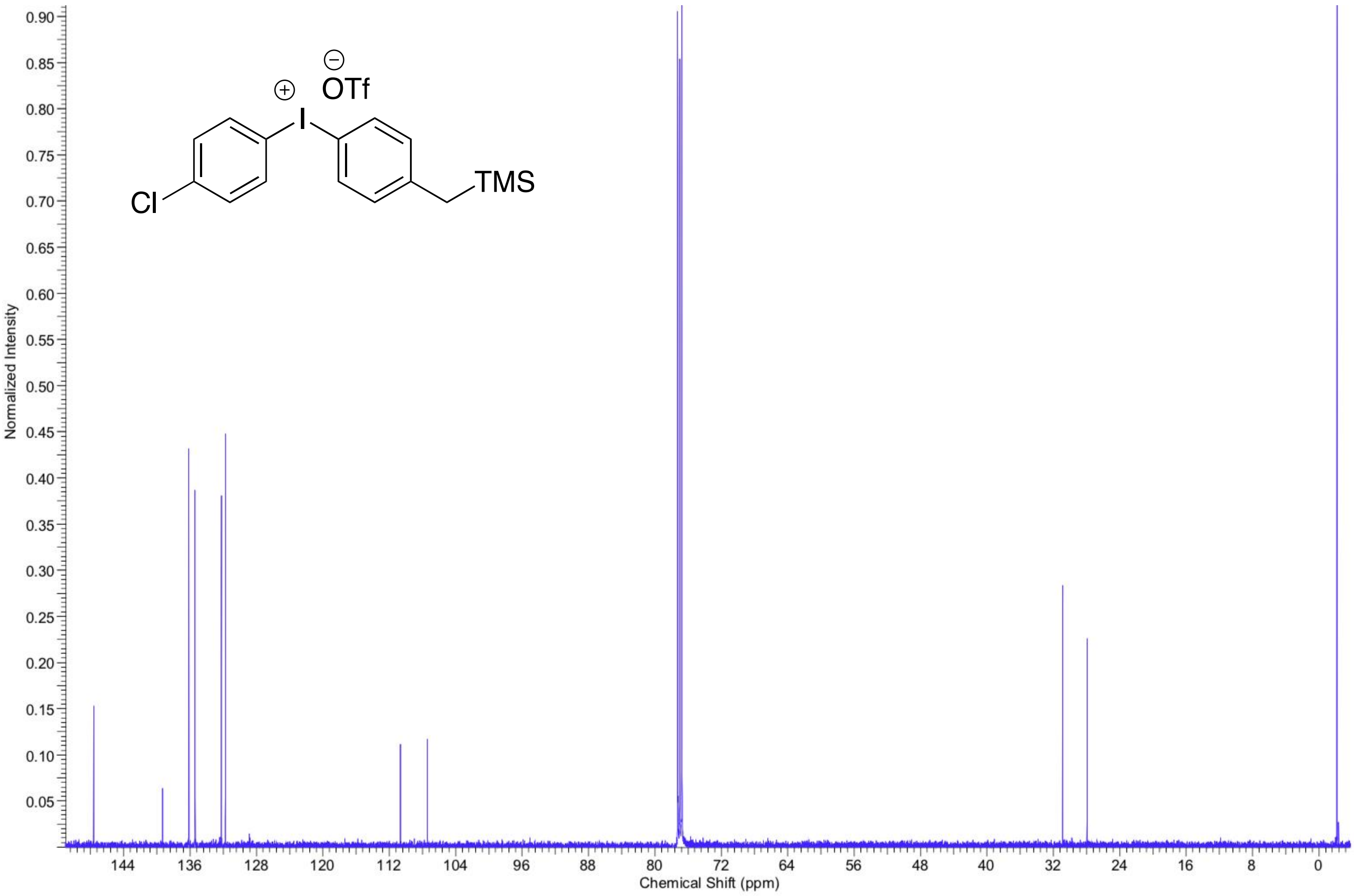

5-Benzyl-2-iodo-1,1'-biphenyl (2e)

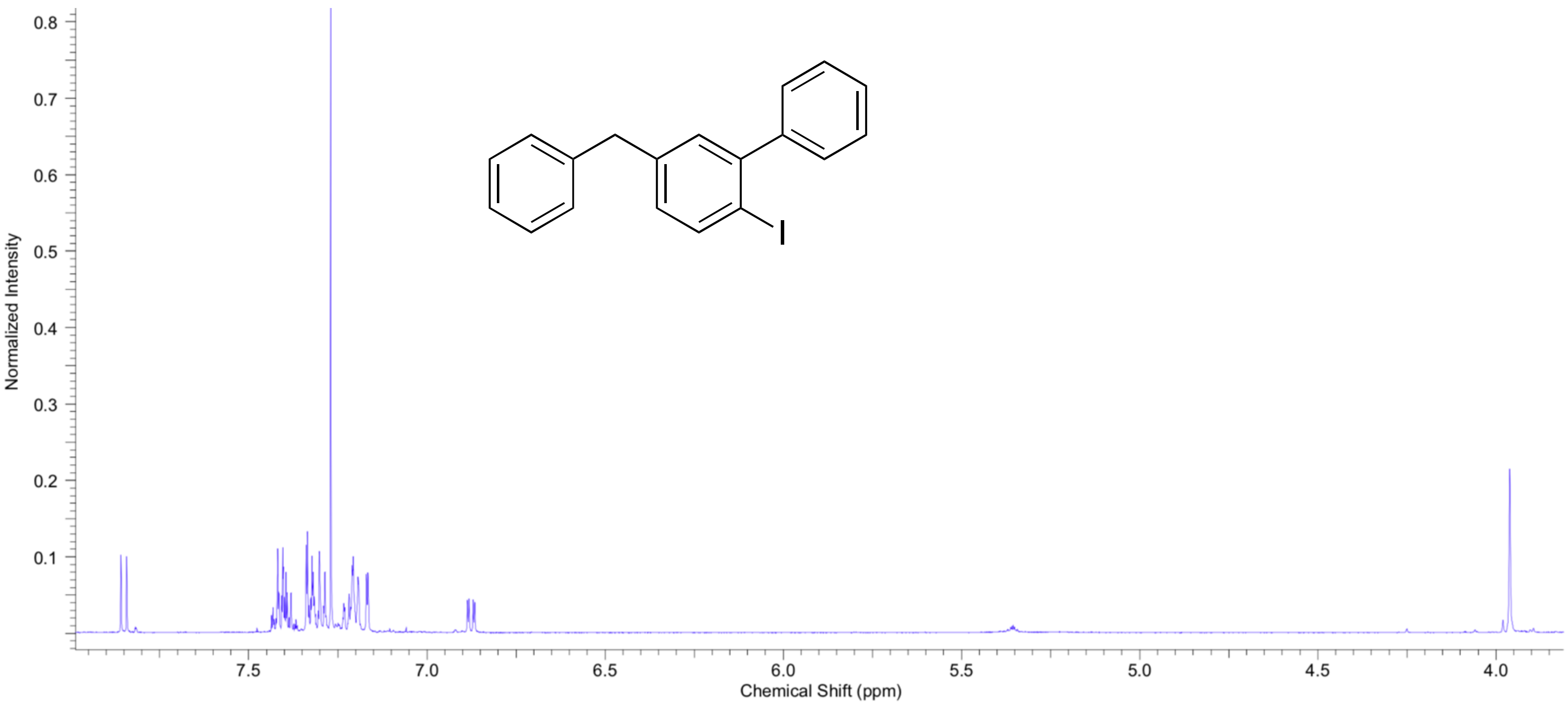

5-Benzyl-2-iodo-1,1'-biphenyl (2e)

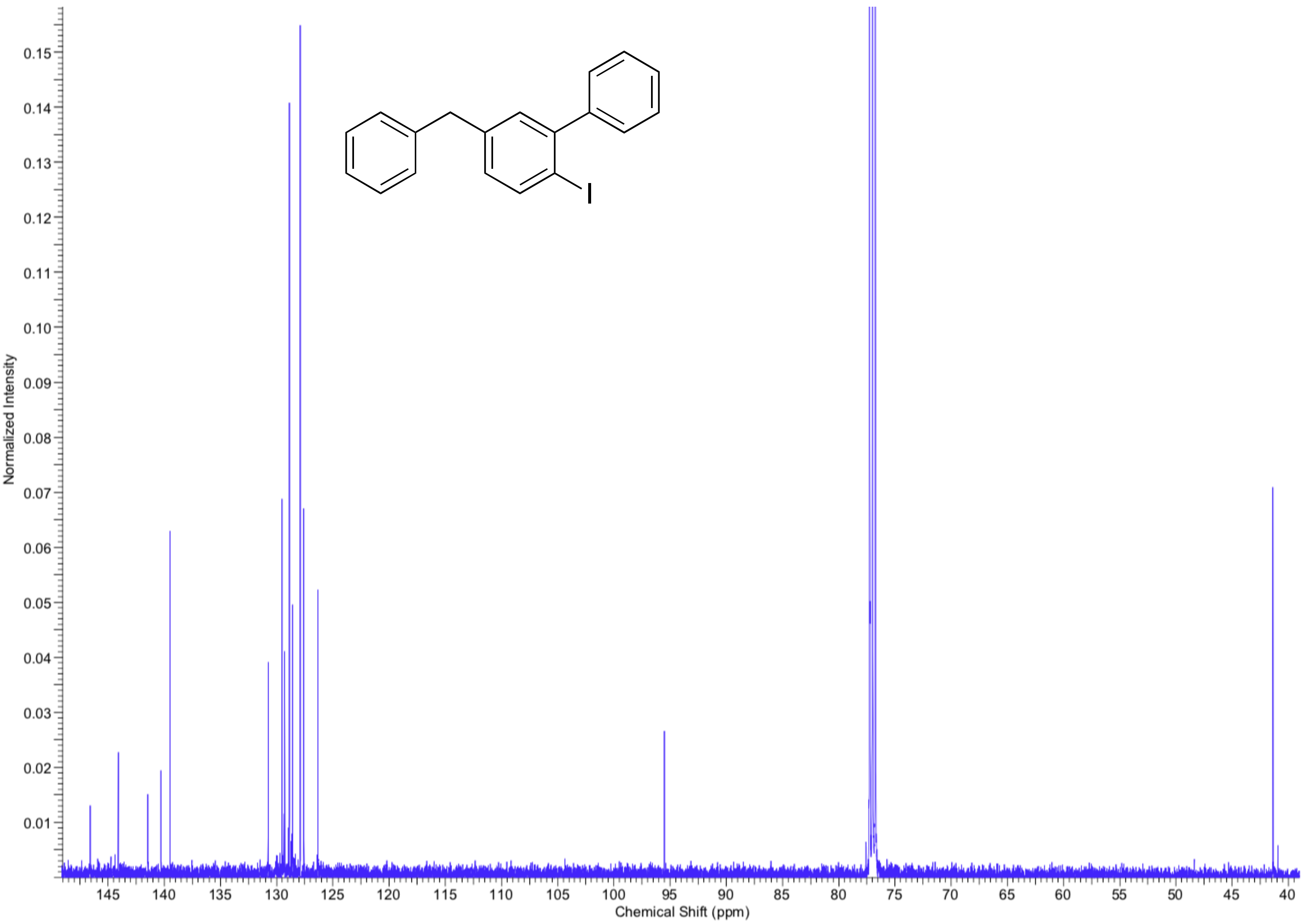

4-Benzyl-1-iodo-2-methylbenzene (2f)

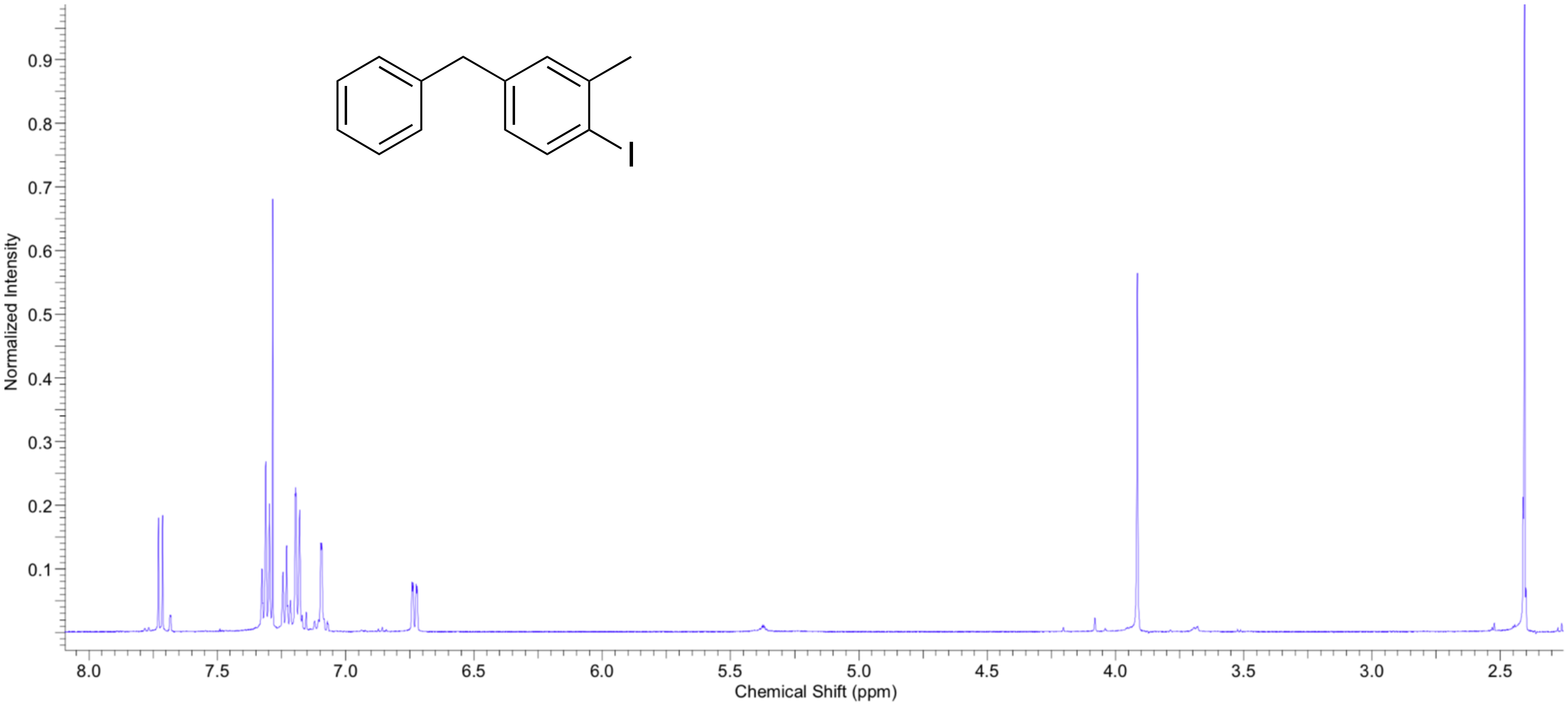

4-Benzyl-1-iodo-2-methylbenzene (2f)

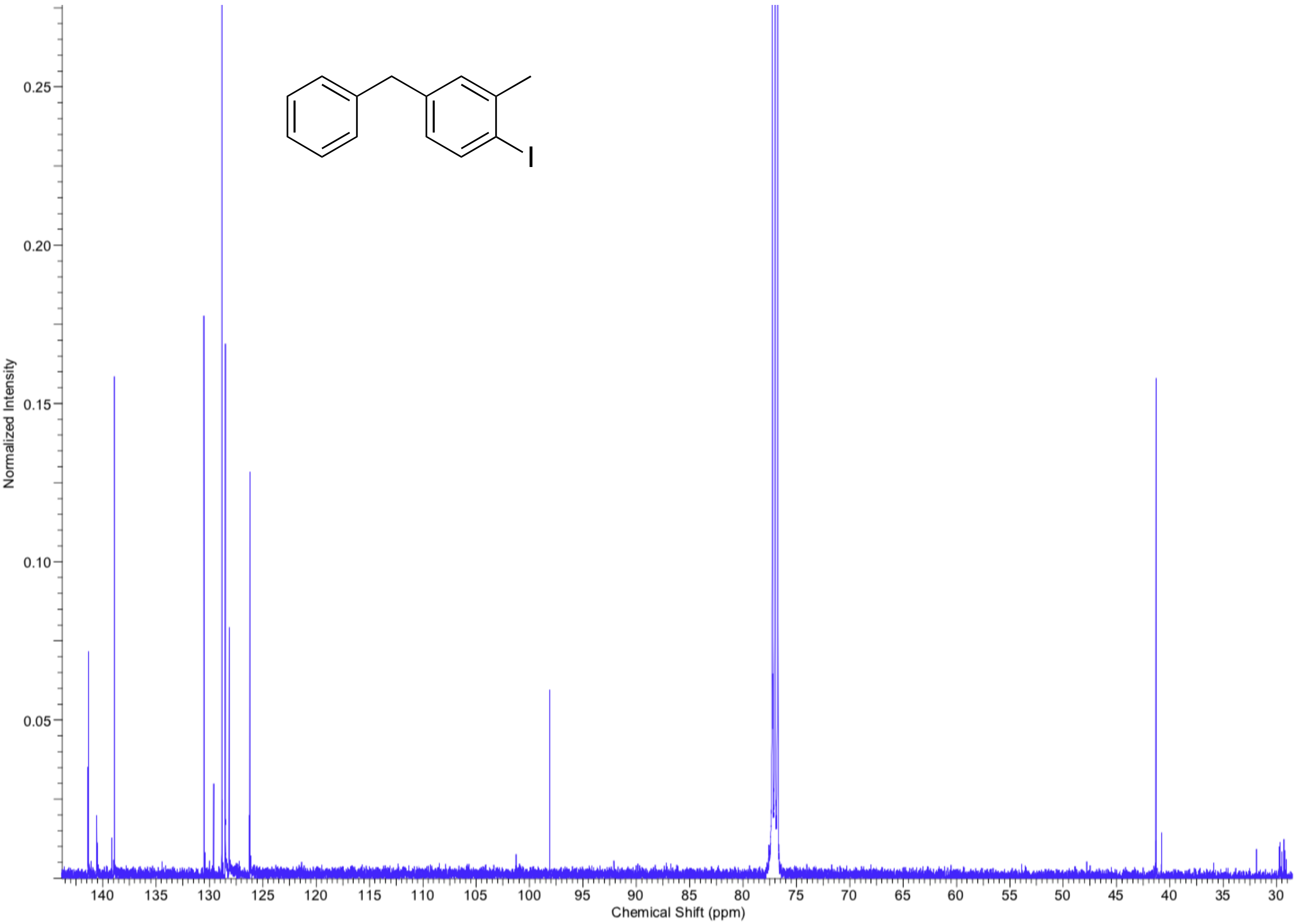

1-Benzyl-4-iodo-2-methylbenzene (2g)

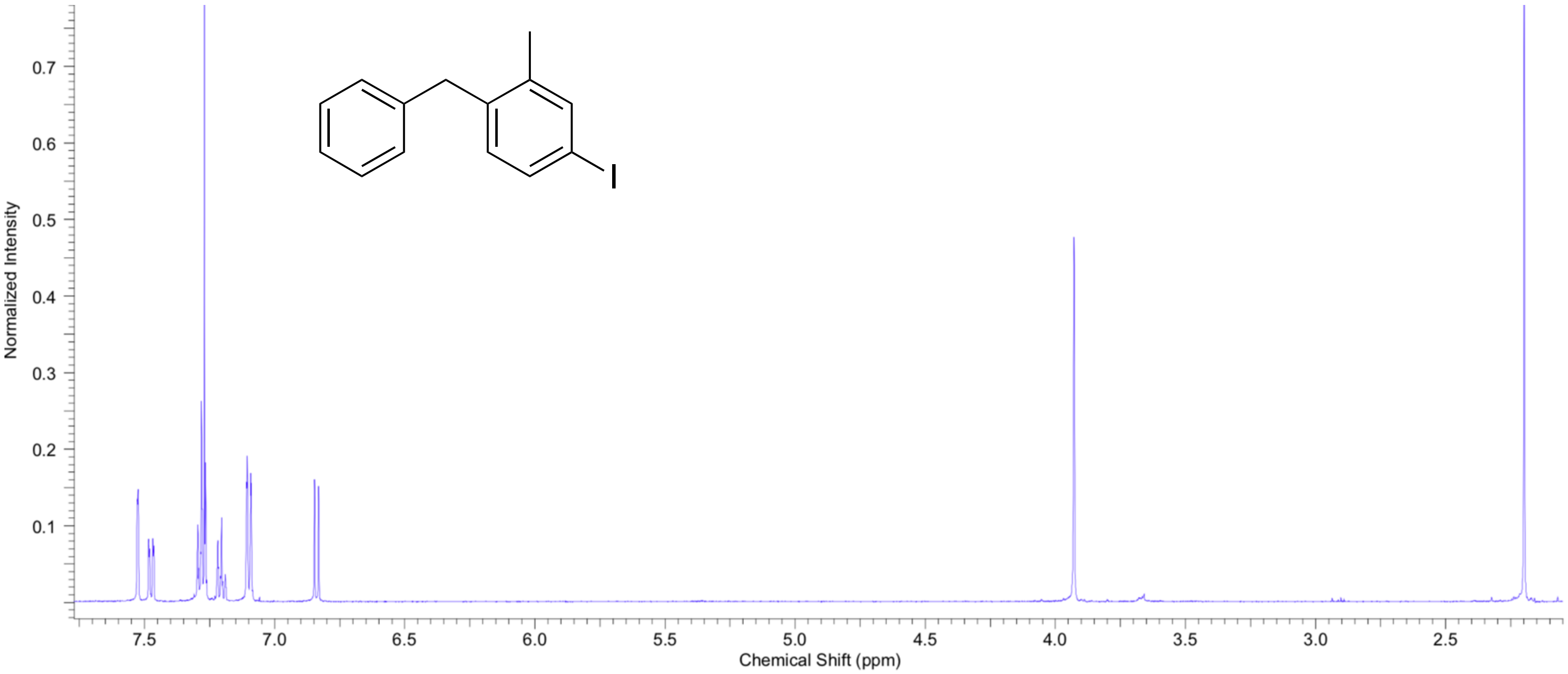

1-Benzyl-4-iodo-2-methylbenzene (2g)

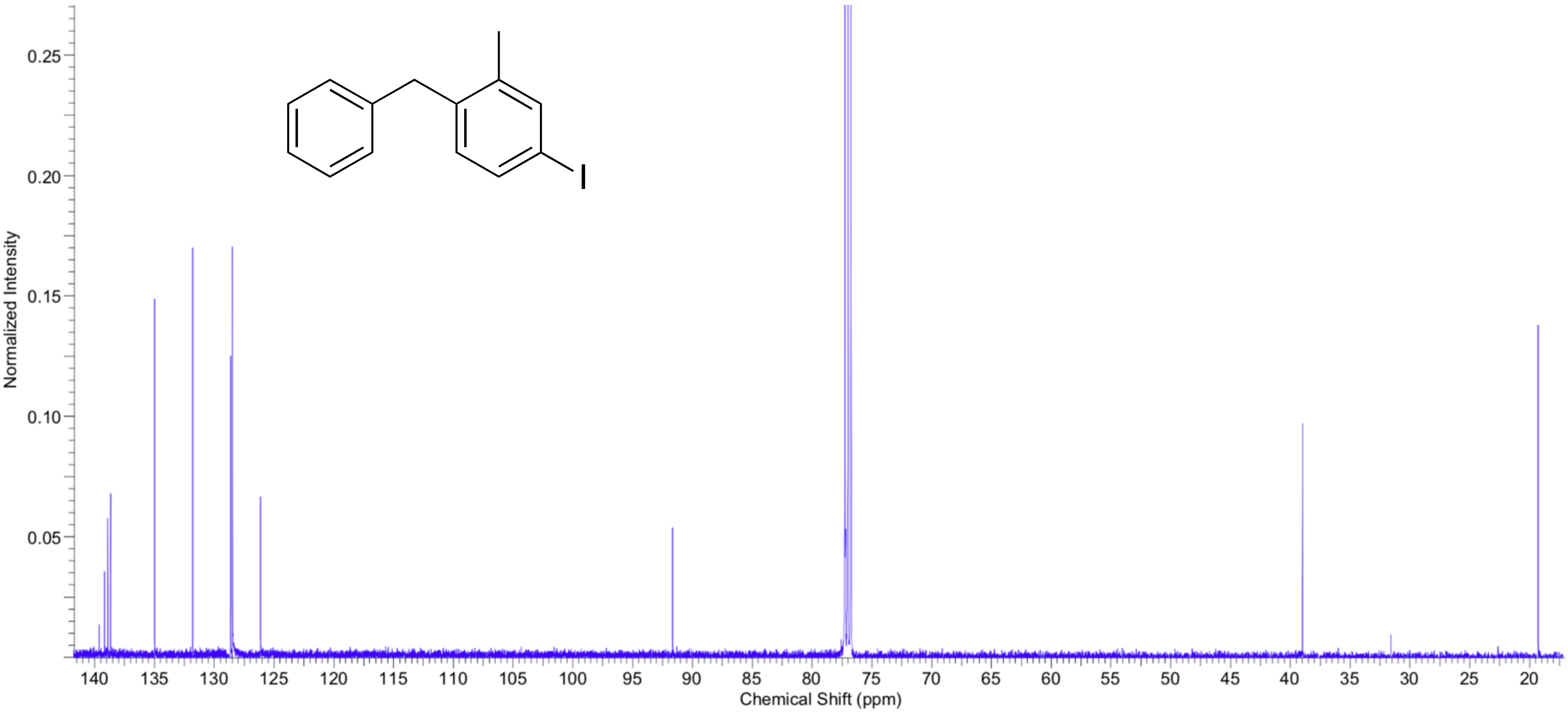

4-Benzyl-2-iodo-1-methoxybenzene (2h)

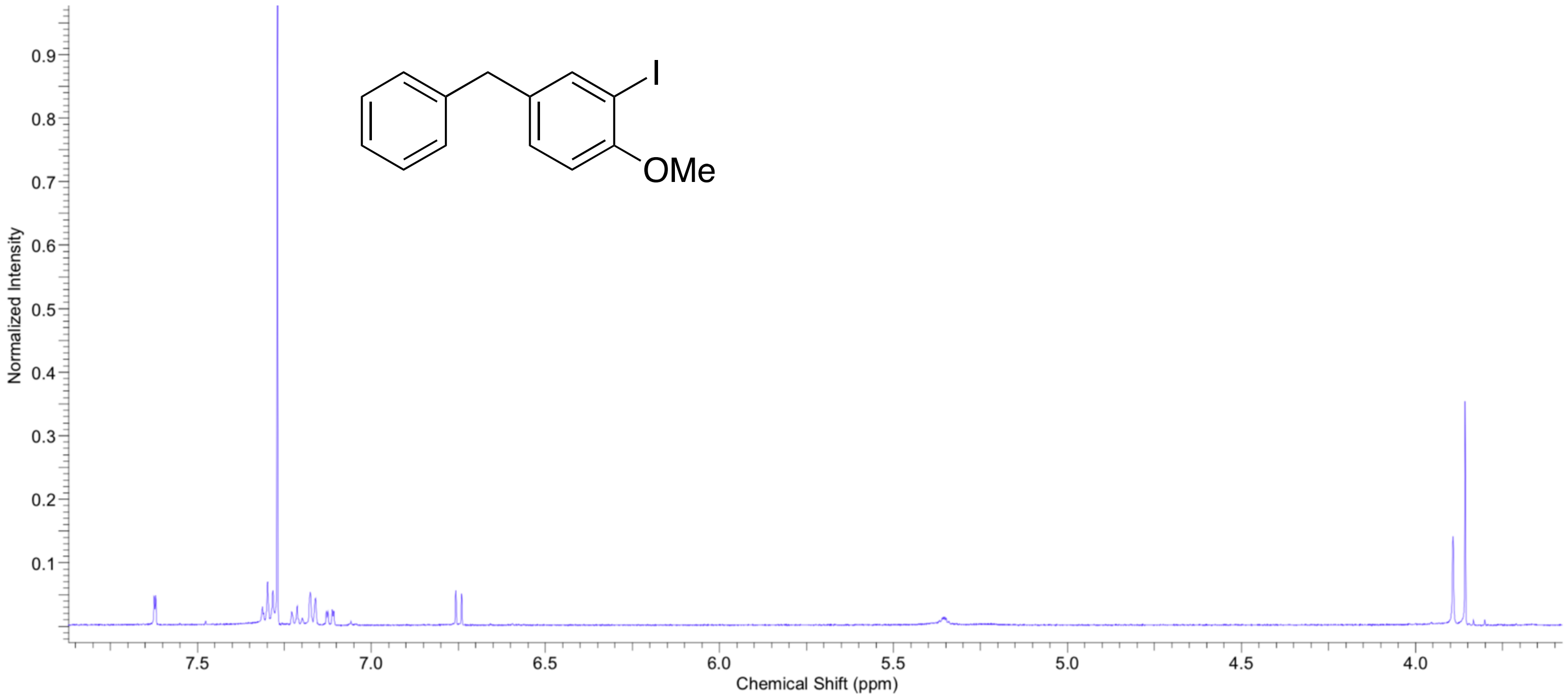

4-Benzyl-2-iodo-1-methoxybenzene (2h)

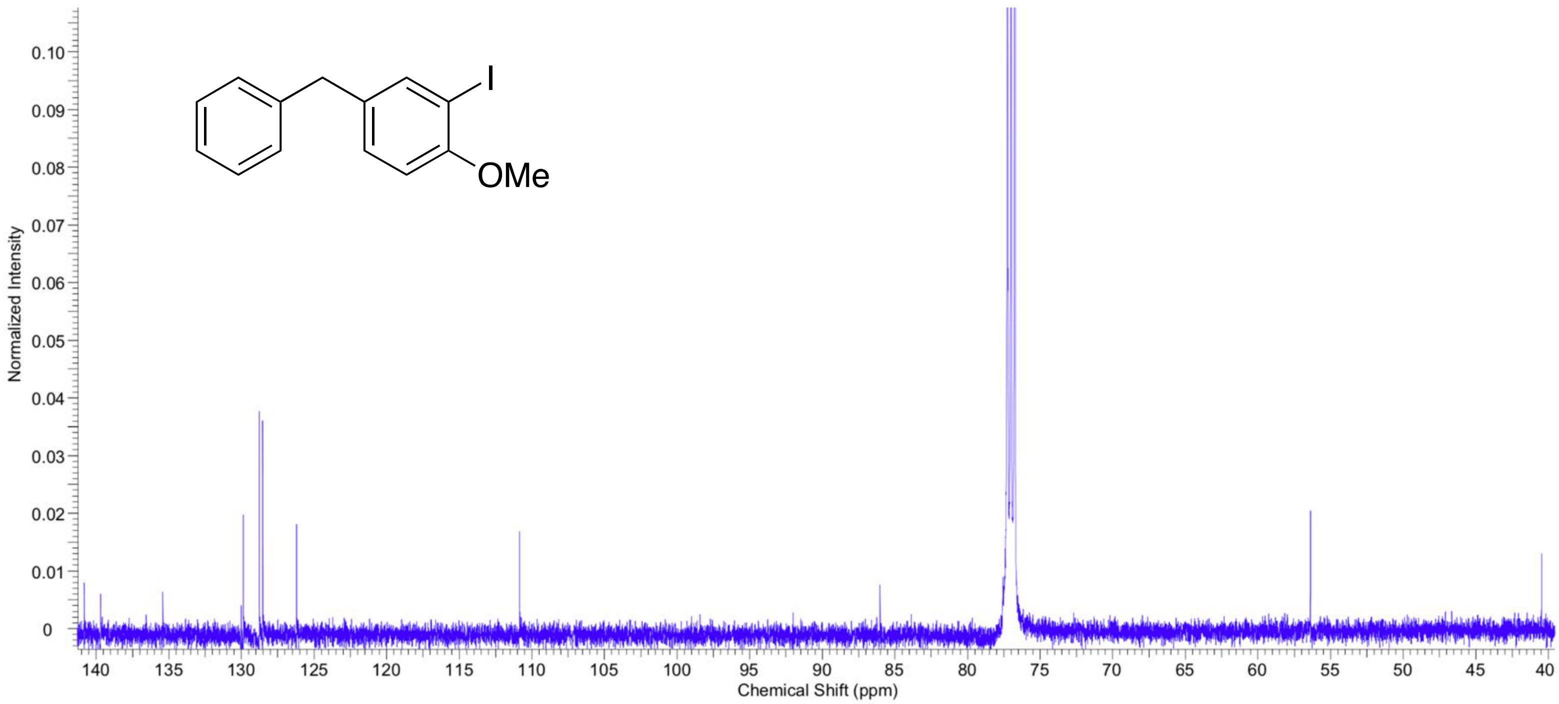

1-Benzyl-2-iodo-4-methoxybenzene (2i)

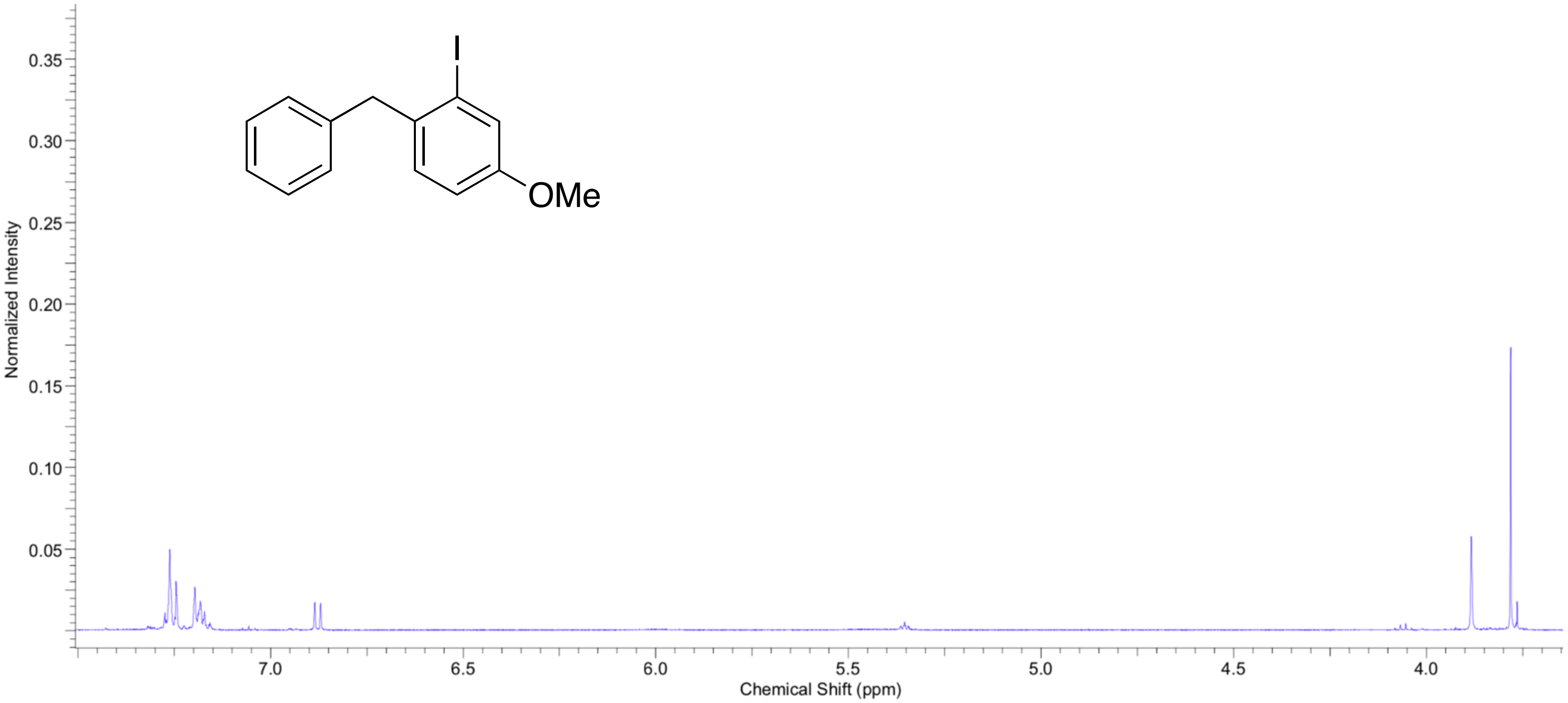

1-Benzyl-2-iodo-4-methoxybenzene (2i)

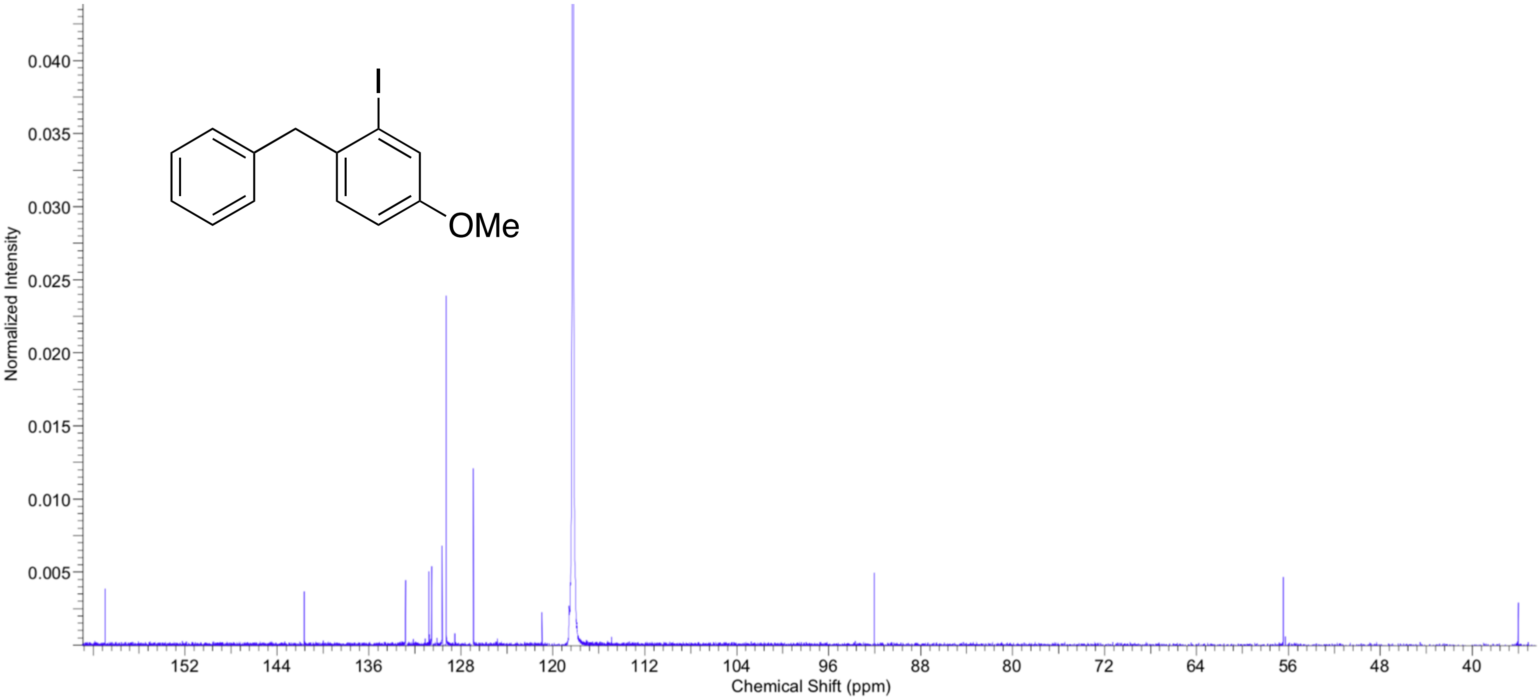

2-Benzyl-5-iodothiophene (2j)

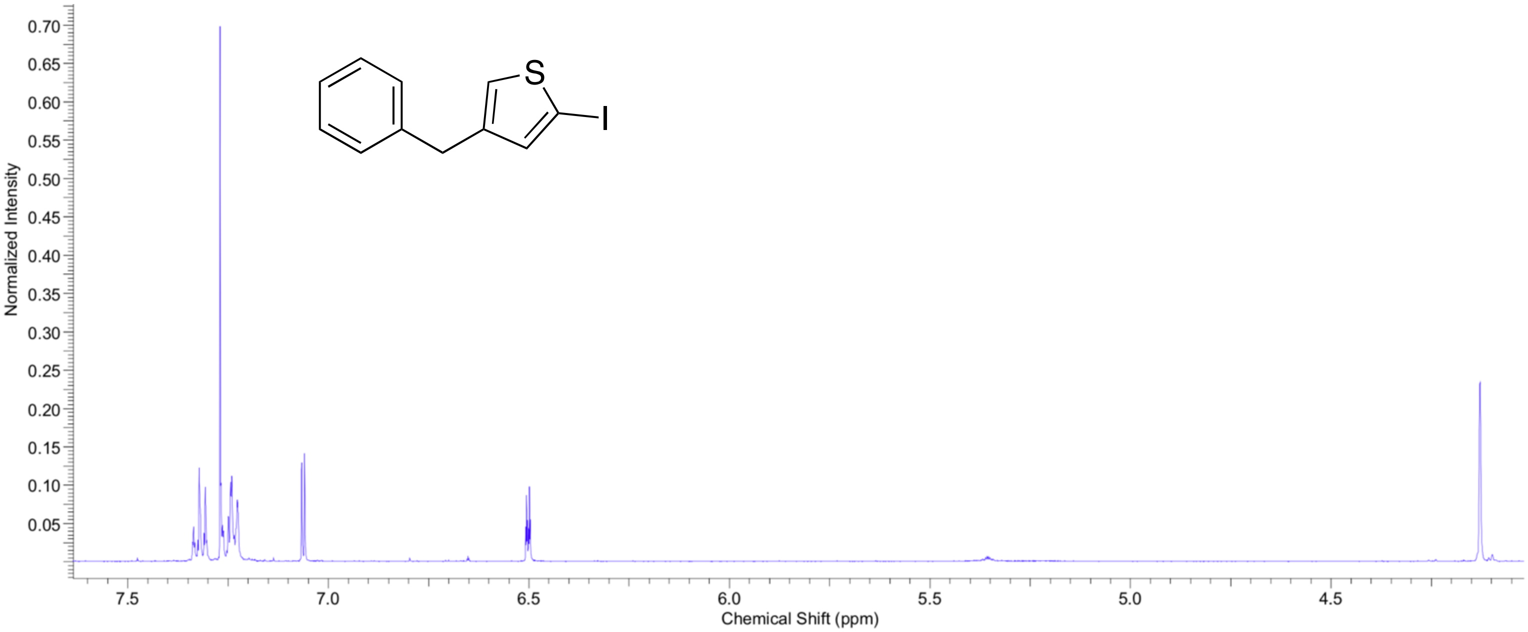

2-Benzyl-5-iodothiophene (2j)

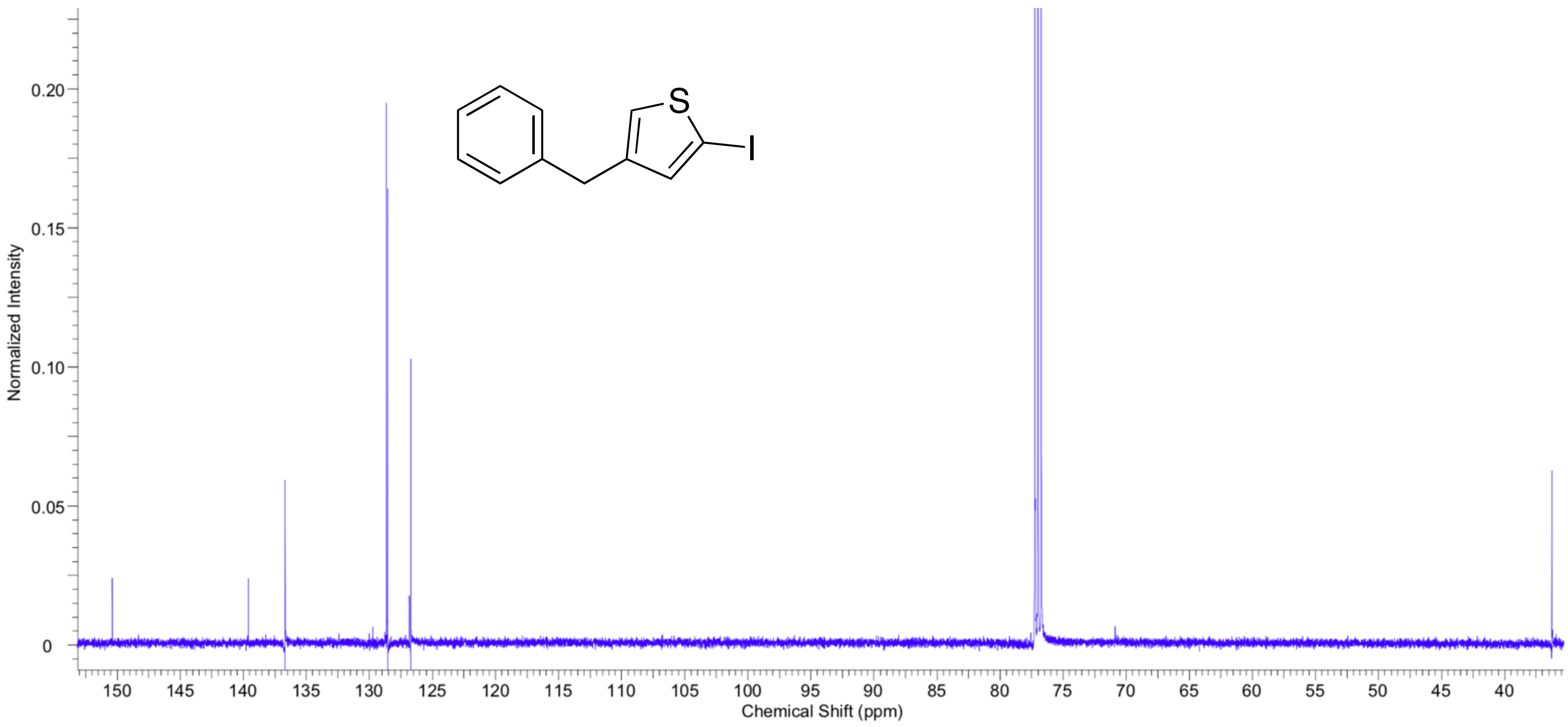

References

1. Haskali, M. B.; Telu, S.; Lee, Y. S.; Morse, C. L.; Lu, S.; Pike, V. W. *J. Org. Chem*. **2016**, 81, 297–302.

2. Kaźmierczak, P.; Skulski, L.; *Synthesis.* **1998,** *12,* 1721-1723.

3. Landge, K. P.; Jang, K. S.; Lee, S. Y.; Chi, D. Y. *J. Org. Chem.* **2012**, *77*, 5705–5713.

4. Graskemper, J. W.; Wang, B.; Qin, L.; Neumann, K. D.; Dimagno, S. G. *Org. Lett.* **2011**, *13*, 3158–3161.

5. Hossain, M. D.; Kitamura, T. *J. Org. Chem.* **2005**, *70*, 6984–6986.

6. Chun, J. H.; Pike, V. W. *J. Org. Chem.* **2012**, *77*, 1931-1938.

7. Iinuma, M.; Moriyama, K.; Togo, H. *Synlett.* **2012,** *23*, 2663−2666.

8. Yu, P.; Zhang, G.; Chen, F.; Cheng, J. *Tetrahedron Lett.* **2012,** *53*, 4588−4590.

9. Liu, W; Yang, X; Gao, Y; Li, C. *J. Am. Chem. Soc.* **2017**, *139*, 8621-8627.

10. Huo, H; Harms, K; Meggers, E. *J. Am. Chem. Soc.* **2016**, *138*, 6936-6939.
